# Where Did the Y Chromosome in the Spiny Rat Go, and How Did It Get There?

**DOI:** 10.1093/molbev/msaf102

**Published:** 2025-05-06

**Authors:** Miki Okuno, Kentaro Matsuoka, Yuta Mochimaru, Takahiro Yamabe, Mayou Okano, Takamichi Jogahara, Atsushi Toyoda, Asato Kuroiwa, Takehiko Itoh

**Affiliations:** Division of Microbiology, Department of Infectious Medicine, Kurume University School of Medicine, Kurume, Fukuoka 830-0011, Japan; School of Life Science and Technology, Institute of Science Tokyo, Tokyo 152-8550, Japan; School of Life Science and Technology, Institute of Science Tokyo, Tokyo 152-8550, Japan; School of Life Science and Technology, Institute of Science Tokyo, Tokyo 152-8550, Japan; School of Life Science and Technology, Institute of Science Tokyo, Tokyo 152-8550, Japan; Faculty of Law, Economics and Management, Okinawa University, Naha, Okinawa 902-8521, Japan; Comparative Genomics Laboratory, National Institute of Genetics, Mishima, Shizuoka 411-8540, Japan; Advanced Genomics Center, National Institute of Genetics, Mishima, Shizuoka 411-8540, Japan; Reproductive and Developmental Science, Biosystems Science Course, Graduate School of Life Science, Hokkaido University, Sapporo, Hokkaido 060-0810, Japan; Division of Reproductive and Developmental Biology, Department of Biological Sciences, Faculty of Science, Hokkaido University, Sapporo, Hokkaido 060-0810, Japan; School of Life Science and Technology, Institute of Science Tokyo, Tokyo 152-8550, Japan

**Keywords:** Y chromosome, comparative genomics, spiny rat, chromosome evolution, chromosome-level genome assembly

## Abstract

The XX/XY sex chromosome system is highly conserved across mammals, with rare exceptions where males lack a Y chromosome. Among these is the genus *Tokudaia*, a group of spiny rats comprising three species with unique sex chromosome systems deviating from the typical XX/XY pattern. While *Tokudaia osimensis* and *Tokudaia tokunoshimensis* have completely lost the Y chromosome, they retain some Y-linked genes on the X chromosome. In contrast, *Tokudaia muenninki* retains large sex chromosomes where both the X and Y chromosomes have fused with an autosome pair, carrying multi-copied Y-linked genes, including *Sry*. In this study, we generated chromosome-level genome assemblies for male individuals of all three *Tokudaia* species. By investigating loci typically associated with rodent Y-linked genes, we characterized sequences derived from the *Tokudaia* Y-chromosomal most recent common ancestor (*Tokudaia* Y-MRCA) and traced their evolutionary trajectories. Our analyses revealed that an initial X-to-Y translocation of a sequence containing the boundary-associated segmental duplication in a common ancestor of *Tokudaia* marked the beginning of their unique sex chromosome evolution. The boundary-associated segmental duplication, uniquely multi-copied in *Tokudaia*, facilitated further rearrangements through nonallelic homologous recombination and duplications. These processes culminated in subsequent Y-to-X translocations and duplications, leading to the complete loss of the Y chromosome as a distinct entity while preserving Y-linked genes in a multicopy state on the X chromosome. These findings highlight *Tokudaia*'s rapid sex chromosome evolution within 3 million years and provide insights into the mechanisms underlying Y chromosome loss, contributing to a broader understanding of sex chromosome evolution in rodents.

## Introduction

The *Sry*-dependent sex determination system, encoded on the Y chromosome, is conserved in most mammals (Sinclair et al. 1990; Koopman et al. 1991; Goodfellow and Lovell-Badge 1993). However, certain mammals, such as *Tokudaia* and *Ellobius*, have atypical sex chromosomes and distinct sex determination systems (Matthey 1953; Honda et al. 1977, 1978). The genus *Tokudaia* includes three species—*Tokudaia muenninki* (Okinawa spiny rat), *Tokudaia osimensis* (Amami spiny rat), and *Tokudaia tokunoshimensis* (Tokunoshima spiny rat)—which are endemic to the islands of Okinawa, Amami Ōshima, and Tokunoshima in southwestern Japan. Phylogenetic studies indicate that *T. muenninki* diverged approximately 2.5 to 2.7 million years ago (Mya) (Murata et al. 2012), with *T. osimensis* and *T. tokunoshimensis* diverging later, approximately 1.1 Mya (Murata et al. 2010). The karyotypes of *T. osimensis* (2n = 25) and *T. tokunoshimensis* (2n = 45) exhibit an XO/XO system, where males lack a Y chromosome and both sexes have only one X chromosome (Honda et al. 1977, 1978; Nakamura et al. 2007). Additionally, the male-determining factor *Sry* is absent in both species (Soullier et al. 1998; Sutou et al. 2001). As a result, *T. osimensis* and *T. tokunoshimensis* utilize a *Sry*-independent sex determination system, prompting investigations into novel mechanisms of sex determination (Kobayashi et al. 2007; Terao et al. 2022). Recent whole-genome analyses of *T. osimensis* revealed a 17-kbp male-specific duplication upstream of *Sox9*, including a region homologous to *enh14* in mice. This duplication was found to promote *Sox9* expression in gene-edited mice; however, no sex reversal occurred, suggesting that it is necessary but not sufficient for male determination, leaving the mechanism unresolved (Terao et al. 2022). Moreover, fluorescence in situ hybridization (FISH) analysis has revealed that some Y chromosome genes commonly found in mammals have been translocated to the distal region of the X chromosome in *T. osimensis* and *T. tokunoshimensis* (Sutou et al. 2001; Arakawa et al. 2002; Kuroiwa et al. 2010). In contrast, *T. muenninki* (2n = 44) has a typical XX/XY karyotype; however, its sex chromosomes have fused with autosomes, creating a large pseudoautosomal region (PAR) (Murata et al. 2010, 2012). RNA-seq analysis indicates that recombination suppression has initiated in the autosome-derived region physically adjacent to the ancestral sex chromosomes, leading to sequence divergence (Murata et al. 2015). Additionally, multiple Y-linked genes, including *Sry*, have undergone multicopy amplification in *T. muenninki*, suggesting that it may possess a unique sex determination mechanism (Murata et al. 2010, 2016). Consequently, *Tokudaia* represents an appealing model for studying chromosomal evolution, especially that of sex chromosomes. However, all three species are endangered (IUCN 2024), limiting the availability of research resources. Previous studies have primarily relied on bacterial artificial chromosome (BAC)-based FISH analyses (Kobayashi et al. 2008; Kuroiwa et al. 2010; Murata et al. 2016), partial genome analysis (Murata et al. 2016), and RNA-seq to identify genes (Murata et al. 2015). The only whole-genome analysis identifying a male-specific region in *T. osimensis* used short-read sequencing, resulting in fragmented X chromosome sequences and restricting comprehensive sex chromosome analysis (Terao et al. 2022). These genome data were later used in the analysis by Li et al. (2024), which confirmed the translocation of several additional Y-linked genes to the X chromosome beyond those already identified by FISH (Sutou et al. 2001; Arakawa et al. 2002; Kuroiwa et al. 2010). However, their study was limited to gene presence confirmation and did not conduct any genome sequence-based analysis. Moreover, the genomes of the other two *Tokudaia* species have not even been sequenced.

In this study, we constructed chromosome-level genome assemblies for male individuals of all three *Tokudaia* species using long-read sequencing and Hi-C technology, enabling a comprehensive comparative genomic analysis of their sex chromosomes. Our primary aim is to establish high-quality genome assemblies and compare their chromosomal structures to understand how the Y chromosome of their common ancestor (the *Tokudaia* Y-chromosomal most recent common ancestor, *Tokudaia* Y-MRCA) has evolved and contributed to shaping the current sex chromosome structures in each species. By investigating these structural changes in the *Tokudaia* lineage, we aim to provide insights into the diversity of sex chromosome evolution in mammals.

## Results

### Genome Construction of the Three *Tokudaia* Species

We constructed chromosome-level genome assemblies for all three *Tokudaia* species using PacBio CLR and Hi-C data, with detailed methodology and statistics provided in *Scientific Data* (Okuno et al. 2023). Unfortunately, the ancestral Y chromosome of *T. muenninki* was highly fragmented, prompting us to acquire additional PacBio HiFi data for reanalysis, which allowed us to achieve chromosome-level assembly for all chromosomes, including the Y chromosome. By reconstructing of the *T. muenninki* genome using HiFi reads, we successfully generated assembly with a N50 of 171.5 Mbp, totaling 3,674.6 Mbp (Table 1), which is approximately 1,014 Mbp longer than the previously assembled genome. This discrepancy is likely due to the inclusion of highly repetitive heterochromatic regions in the assembly. The estimated genome size is 3,362.6 Mbp, and when accounting for the half-coverage of the sex chromosomes, this result demonstrates good concordance. Notably, this assembly successfully resolved the highly fragmented Y chromosome from the previous assembly and reconstructed the neo-ancX chromosome as a single sequence, including the heterochromatic region. A summary of the genome assembly results for all three species is provided in Table 1. As shown in Table 1 and supplementary fig. S19, Supplementary Material online our assemblies exhibit high continuity and completeness, as evaluated by BUSCO gene assessment. However, independent experimental validation of the assembly has not been conducted.

**Table 1:** Assembly statistics of the three *Tokudaia* species genome**s**

	*T. osimensis*	*T. tokunoshimensis*	*T. muenninki*
	2n = 25, XO/XO	2n = 45, XO/XO	2n = 44, XX/XY
Genome assembly statistics			
#Scaffolds	123	159	34
#Chromosome-level scaffolds	13	23	23
#Unplaced scaffolds	110	136	11
Total scaffold length (bp)	2,445,255,239	2,477,294,292	3,674,585,994
Total Chr.-level scaffold length (bp)	2,440,098,537	2,473,996,358	3,651,392,569
Anchored to chromosome (%)	99.79	99.87	99.37
Longest scaffold (bp)	269,377,760	179,766,896	346,715,556
Contig N50 (bp)	8,462,634	13,597,559	138,437,150
Contig L50	88	57	11
Scaffold N50 (bp)	234,036,378	125,076,325	171,451,252
Scaffold L50	5	9	8
Gaps (bp)	270,541	205,000	9,500
BUSCO evaluation (v5.7.1, genome mode, glires_odb10)			
Complete BUSCOs (%)	98.72	98.74	98.80
Single-copy BUSCOs (%)	98.15	97.99	97.96
Duplicated BUSCOs (%)	0.57	0.75	0.84
Fragmented BUSCOs (%)	0.70	0.72	0.67
Missing BUSCOs (%)	0.57	0.54	0.52

**Fig. 1.**
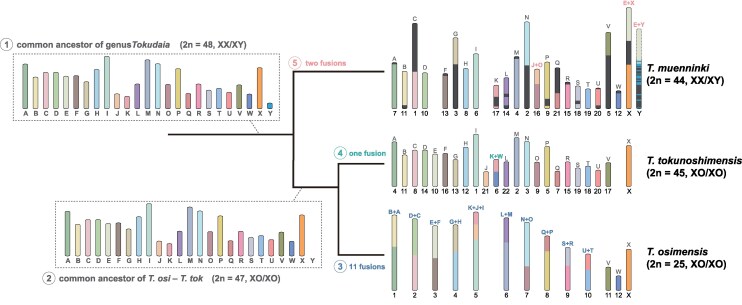
Evolutionary transitions from ancestral chromosomes of the three *Tokudaia* species. Evolutionary transitions from ancestral chromosomes to the current karyotypes of the three species, based on synteny analysis. The 25 ancestral chromosomes of the common ancestor (labeled A to Y, where A to W are autosomes, and X/Y are sex chromosomes) are shown, along with the fusion events between corresponding chromosomes. The common ancestor of the three species is estimated to have a karyotype of 2n = 48 (XX/XY) (1). After divergence, the common ancestor of *T. osimensis* and *T. tokunoshimensis* lost the Y chromosome, leading to a 2n = 47 (XO/XO) karyotype (2). Subsequent divergence led to 11 chromosomal fusions in *T. osimensis*, resulting in the current karyotype of 2n = 25 (XO/XO) (3), and one chromosomal fusion in *T. tokunoshimensis*, leading to the current 2n = 45 (XO/XO) karyotype (4). In contrast, *T. muenninki* experienced two chromosomal fusions, including a fusion between autosome E and a sex chromosome (5), resulting in 2n = 44 (XX/XY).

Subsequently, we analyzed the synteny relationships among the three species and predicted their ancestral karyotypes (Fig. 1, supplementary figs. S1 and S2, Supplementary Material online). Synteny in both autosomes and the X chromosome was highly conserved across species, with karyotype differences attributable to chromosomal fusions. The common ancestral karyotype likely comprised 23 autosomes and sex chromosomes (2n = 48), whereas the common ancestors of *T. osimensis* and *T. tokunoshimensis* likely lost the Y chromosome, resulting in a 2n = 47 karyotype. All chromosomal fusions appeared to have occurred in a species-specific manner: *T. osimensis* has 12 autosomes and one X chromosome resulting from 11 chromosomal fusions; *T. tokunoshimensis* has 22 autosomes and one X chromosome because of one fusion; and *T. muenninki* has 21 autosomes and sex chromosomes resulting from two fusions, one involving a sex chromosome and the other an autosome.

### Genomic Overview of the *Tokudaia* Y-MRCA-Originated Locus in the Three *Tokudaia* Species

**Fig. 2.**
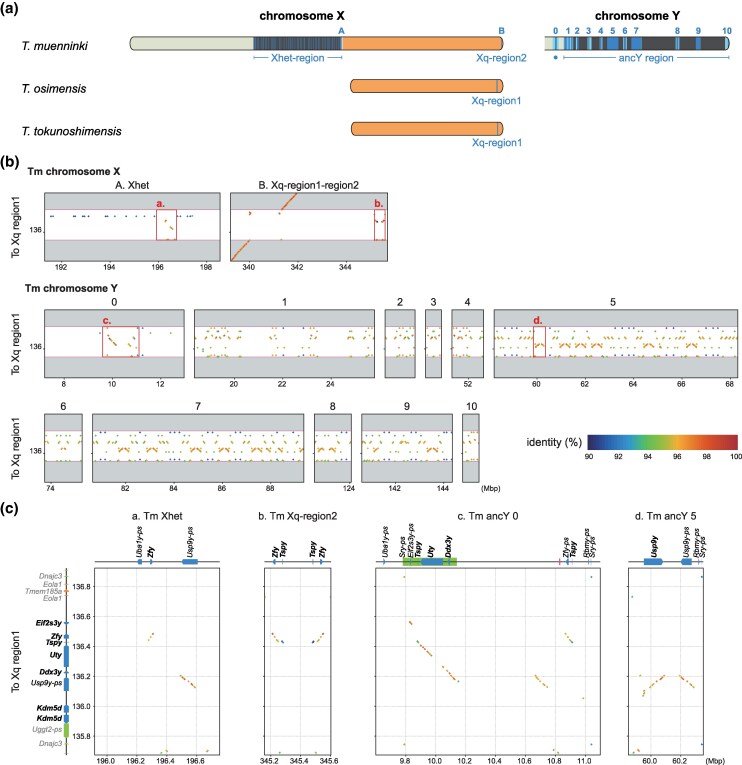
*Tokudaia* Y-MRCA-derived regions in the three species. a) Representation of chromosomal regions containing *Tokudaia* Y-chromosomal most recent common ancestor (Y-MRCA)-derived sequences in the three species. In *T. muenninki*, two regions were identified on the X chromosome (Xhet-region, Xq-region2) and one large region on the Y chromosome (ancY-het-region). In both *T. osimensis* and *T. tokunoshimensis*, a single region was identified on the X chromosome (Xq-region1). b) Dot-plot comparison of Y-MRCA-derived regions between *T. osimensis* (Xq-region1 and its surrounding regions) and *T. muenninki* (Y-MRCA-derived regions). In *T. muenninki*, the corresponding X chromosomal regions are: A) the 3′ terminal region of the Xhet-region and B) the region spanning Xq-region1 to Xq-region2, while the Y chromosomal regions correspond to blocks 0 to 10. The specific positions of these regions are indicated in a). Strong synteny, visualized as continuous diagonal lines, is observed between upstream and downstream regions of *T. osimensis* Xq-region1 and the corresponding Xq-region1 in *T. muenninki*. In contrast, in the Y-derived regions, synteny is fragmented, appearing as sparse and short diagonal lines, indicating disrupted structural conservation. c) Magnified views of the regions outlined by squares in b). The region labels (a to d) correspond to those indicated in b). The figure shows that the longest syntenic segment between *T. osimensis* Xq-region1 and *T. muenninki* is within block 0 of ancY, spanning approximately 400 kb, where three genes (*Ddx3y*, *Uty*, and *Tspy1*) are retained in the same order. Beyond this region, only scattered instances of two-gene synteny are observed.

In contrast to other chromosomes—such as the X chromosome shown as an example in supplementary fig. S3a, Supplementary Material online—no chromosome-scale synteny, or even synteny at the megabase level, was detected in the Y chromosome due to its extensive structural rearrangements and differentiation. To identify regions derived from *Tokudaia* Y-MRCA, we searched for known rodent Y-linked genes across the whole genome sequences. The copy numbers and chromosomal locations of the identified Y-linked genes are summarized in Table 2. As shown in Fig. 2a, in *T. osimensis* and *T. tokunoshimensis*, these genes were detected at a single locus in the distal Xq region (Xq-region1). In contrast, in *T. muenninki*, they were distributed across three distinct regions: the heterochromatic region between the neo-sex and ancestral X chromosome segments (Xhet-region, previously classified as a heterochromatic region based on cytogenetic studies; Murata et al. 2010; Murata et al. 2012), the distal Xq region (Xq-region2), and around the ancestral Y region (ancY-het-region). Xq-region2 is distinct from Xq-region1 and originated from an independent translocation event. Before delving into the details of each locus, we first provide an overview of these identified Y-MRCA-derived regions. The structural differences among these loci are striking. Xq-region1 in *T. osimensis* and *T. tokunoshimensis* spans approximately 900 kb to 1 Mb, whereas Xq-region2 in *T. muenninki* consists of duplicated 150 kb segments totaling around 300 kb. In contrast, the Xhet-region and ancY-het-region in *T. muenninki* are much larger, spanning 80 to 100 Mb, though they are rich in repetitive sequences. Despite the relatively short divergence time of approximately 2.5 million years between *T. muenninki* and the other two species, extensive structural rearrangements and repeat insertions have independently occurred in each lineage. As a result, while small-scale synteny can be detected at certain loci, no large-scale synteny is observed (supplementary fig. S3b, Supplementary Material online and Fig. 2b). The highest degree of synteny at the gene order level is observed between Xq-region1 in *T. osimensis*/*T. tokunoshimensis* and the proximal part of ancY-het-region in *T. muenninki* (Fig. 2c-c), where three genes (*Ddx3y*, *Uty*, and *Tsp1y*) are retained in the same order within a region of approximately 350 kb. Beyond this, only scattered instances of two-gene synteny are detected (Fig. 2c-a, b, and d). Given these substantial differences in genomic structure and scale, direct comparisons between the former Y-derived loci of *T. muenninki* and those in *T. osimensis*/*T. tokunoshimensis* are challenging. Moreover, no closely related outgroup species with a fully assembled Y chromosome exists, making ancestral state reconstruction difficult.

**Table 2:** Comparison of Y-linked gene numbers across *Tokudaia* Y-MRCA-derived regions and other rodent species reported to have atypical sex chromosome**s**

	*T. osimensis*		*T. tokunoshimensis*		*T. muenninki*								*M. oregoni* ^a^		*E. lutescens* ^b^	*E. talpinus* ^b^
	(XO/XO)		(XO/XO)		(XX/XY)								(X^M^O/X^P^X^M^)		(XO/XO)	(XX/XX)
	Xq-region1		Xq-region1		Xhet-region		Xq-region1		Xq-region2		ancY-het-region		Male X^P^	Female X^M^	X	X
	Intact	Pseudo	Intact	Pseudo	Intact	Pseudo	Intact	Pseudo	Intact	Pseudo	Intact	Pseudo			Intact/pseudo	Intact/pseudo
*Ddx3y*	1	…	1	…	…	…	…	…	…	…	1	…	o	o	…	…
*Uty*	1	…	1	…	…	…	…	…	…	…	1	…	o	o	…	…
*Eif2s3y*	1	…	1	…	…	…	…	…	…	…	11	2	o	o	1	1
*Uba1y*	…	…	…	…	…	53	…	…	…	…	…	8	?	?	…	…
*Usp9y*	…	1	…	1	…	1	…	…	…	…	7	24	?	?	1	…
*Sry*	…	…	…	…	…	…	…	…	…	…	5	58	o	o	…	…
*Rbmy*	…	…	…	…	…	…	…	…	…	…	5	40	o	o	…	…
*Tspy*	1	…	1	…	…	…	…	…	2	…	62	35	…	…	…	…
*Zfy*	1	…	1	…	1	…	…	…	2	…	15	78	?	?	1	1
*Kdm5d*	2	…	…	1	…	…	…	…	…	…	…	…	o	o	…	…
BASD	3	…	4	…	1	…	2	…	…	2	105	…	…	…	…	…

? Not all exons found.

^a^ Couger et al. (2021).

^b^ Mulugeta et al. (2016).

### *Tokudaia* Y-MRCA-Originated Locus in *T. osimensis* and *T. tokunoshimensis* Genomes

**Fig. 3.**
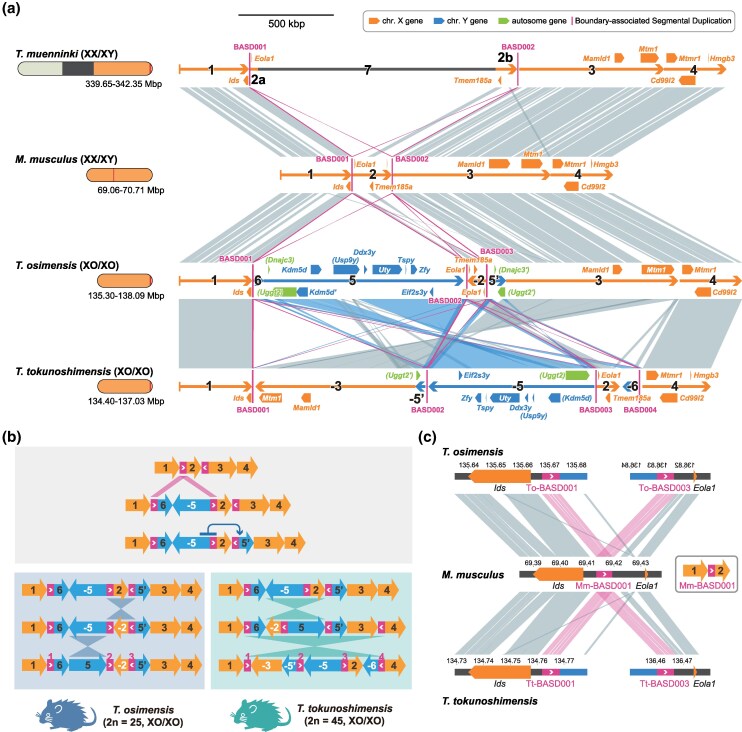
*Tokudaia* Y-MRCA-derived Xq-region1 in *T. osimensis* and *T. tokunoshimensis.* a) Synteny analysis of Xq-region1 and surrounding areas in *T. osimensis* and *T. tokunoshimensis*, alongside corresponding regions in *T. muenninki* and mouse. Seven synteny blocks (SB1 to SB7) were identified among the four species, with alignment lines connecting homologous regions. Chromosome origins are distinguished by fill patterns and labels: X chromosome-derived regions are labeled SB1–SB4, Y chromosome-derived regions are SB5, SB6, and SB5′, and autosomal regions are also indicated. Boundary-associated segmental duplications (BASDs) are shown at the edges of synteny boundaries. SB1 to SB4 on the mouse X chromosome are considered ancestral, while SB5, SB6, and SB5′ (a duplicated portion of SB5, potentially originating from *Tokudaia* Y-MRCA with some autosomal contributions), were inserted into the Xq-region1 of *T. osimensis* and *T. tokunoshimensis*. In *T. muenninki*, SB7, containing repeated sequences similar to those downstream of SB4, was inserted in this region. Structural variations, including inversions, were observed between *T. osimensis* and *T. tokunoshimensis*, particularly in SB5, SB6, SB2, and SB3. b) A schematic diagram of the evolutionary events following the insertion of SB5 and SB6 (derived from *Tokudaia* Y-MRCA) in *T. osimensis* and *T. tokunoshimensis*, based on sequence duplication traces and the most parsimonious prediction of structural variation. Initially, the common ancestor underwent the insertion of SB5 and SB6 (in reverse orientation) between SB1 and SB2 via BASD-mediated NAHR. Subsequently, SB5 and BASD were duplicated and translocated, forming SB5′. After species divergence, each lineage experienced two inversion events, shaping the current genome structures of both species. c) Close-up view of the boundary regions where the insertions likely occurred, using the mouse genome as a reference. Diagonal alignment lines with the mouse genome indicate that the insertion was mediated by the BASD, located between SB1 and SB2 in the mouse genome. The alignments of regions flanking Mm-BASD001, along with their corresponding positions, further confirm the BASD-mediated insertion.

As mentioned above, regions likely derived from *Tokudaia* Y-MRCA in both *T. osimensis* and *T. tokunoshimensis* were located at the same distal X chromosome locus, with an inversion observed between the two species (Fig. 3a). This locus contains seven Y-linked genes (*Zfy*, *Kdm5d*, *Eif2s3y*, *Uty*, *Tspy*, *Ddx3y*, and *Usp9y*) and was the only region in both species that retained intact Y-linked genes. Otherwise, only a previously reported *Rbmy* processed pseudogene was found on an autosome (supplementary fig. S4, Supplementary Material online) (Kuroiwa et al. 2010).

Next, we compared the genomes of both species with the X chromosomes of *T. muenninki* and mice, which lack the insertion of Y-derived sequences in this locus. We defined synteny blocks (SBs) and predicted the evolutionary events that occurred among the four species. Consequently, the region surrounding this locus was divided into seven SBs (Fig. 3a, supplementary fig. S5 and table S1, Supplementary Material online). SB1 to 4 were X chromosome-derived sequences common to all four species, with their order and orientation in mice considered ancestral (supplementary fig. S6, Supplementary Material online). Meanwhile, SB5, SB5′, and SB6 were found only in *T. osimensis* and *T. tokunoshimensis*, indicating an insertion between SB1 and SB4 in their common ancestor. Interestingly, SB5 contains not only Y-linked genes but also autosomal-derived *Uggt2* and *Dnajc3* genes (supplementary fig. S7, Supplementary Material online). As intact copies of these genes are present on autosomes, a genomic region containing parts of these genes is duplicated and translocated. SB5′ (99.2 kbp in *T. osimensis*, 61.3 kbp in *T. tokunoshimensis*) is a duplicated sequence of part of the *Uggt2* and *Dnajc3* regions within SB5, likely duplicated after insertion into their common ancestor. It is uncertain whether these sequences first integrated into the ancestral Y before translocating to the X or were directly inserted into the ancestral X. SB7, found only in *T. muenninki*, is a repeat-rich region homologous to repetitive sequences downstream of SB4 in *T. osimensis* and *T. tokunoshimensis* and is inserted within SB2 (supplementary fig. S5b, Supplementary Material online).

To understand how the genomic structure of the Xq-region1 in both species arose, we conducted a detailed analysis to infer the most plausible sequence of genomic rearrangements. Given the extensive structural variations observed in *Tokudaia* Y-MRCA-derived regions, resolving these recent events provides valuable insights into the dynamics of Y-derived sequences in this lineage. By estimating the most parsimonious insertion positions and arrangements of SB5 and SB6 to minimize the number of inversions in both species (supplementary fig. S8a, Supplementary Material online), we identified two possible scenarios, both suggesting that SB5 and SB6 were inserted between SB1 and SB2 (supplementary fig. S8b, Supplementary Material online). Among these, the scenario depicted in Fig. 3b (supplementary fig. S8b, Supplementary Material online, right) was found to be the most likely. This conclusion is supported by sequence analysis of *T. osimensis*, which revealed that the SB5′ likely originated from SB5 through an inverted duplication event mediated by a shared sequence at its boundaries (supplementary fig. S9, Supplementary Material online). Additionally, the arrangement of homologous sequences around SB5 and SB5′ suggests that an inversion occurred within SB5 after this duplication event. Further details on these rearrangement scenarios can be found in supplementary fig. S9, Supplementary Material online.

Chromosomal rearrangements often leave identifiable sequence features at their junctions, providing insights into their underlying mechanisms. Given that SB5 and SB6 were inserted at the boundary between SB1 and SB2—specifically between the genes *Ids* and *Eola1*—in both *T. osimensis* and *T. tokunoshimensis*, we examined this region to determine whether it harbored any characteristic sequences. Our analysis identified a 6.7 kbp sequence shared between the 3′ end of SB1 and the 5′ end of SB2, exhibiting over 98% sequence identity in *T. osimensis*. This sequence exceeds the commonly used criteria for segmental duplications (SDs; >1 kb length and >90% sequence identity) (Bailey and Eichler 2006). Based on these characteristics, we refer to this sequence as the boundary-associated segmental duplication (BASD; supplementary table S2, Supplementary Material online) throughout the manuscript. Although details of BASD are discussed in a later section, we note here that in common rodents, including *Mus musculus* and *T. muenninki*, BASD is typically present in up to two copies within the Xq-region1 locus. However, in *T. osimensis* and *T. tokunoshimensis*, the copy number has increased to three and four, respectively. Specifically, In *M. musculus* and *T. muenninki*, Mm-BASD001 and Tm-BASD001 are located at the SB1–SB2 boundary, and Mm-BASD002 and Tm-BASD002 are located at the SB2–SB3 boundary. In *T. osimensis*, To-BASD001 is found at the SB1–SB6 boundary, To-BASD002 at the SB5–SB2 boundary, and To-BASD003 at the SB2–SB5′ boundary. In *T. tokunoshimensis*, Tt-BASD001 is located at the SB1–SB3 boundary, Tt-BASD002 at the SB5′–SB5 boundary, Tt-BASD003 at the SB5–SB2 boundary, and Tt-BASD004 at the SB6–SB4 boundary (Fig. 3a). The BASD corresponding to Mm-BASD001 likely facilitated the insertion of SB5 and SB6 via nonallelic homologous recombination (NAHR; Fig. 3c). Interestingly, the BASD was also found at the SB2–SB5′ boundary in *T. osimensis* and at the SB5′–SB5 and SB6–SB4 boundaries in *T. tokunoshimensis*, indicating its role in further structural changes post-insertion (Fig. 3a).

### *Tokudaia* Y-MRCA-Originated Locus in *T. muenninki* Genome

**Fig. 4.**
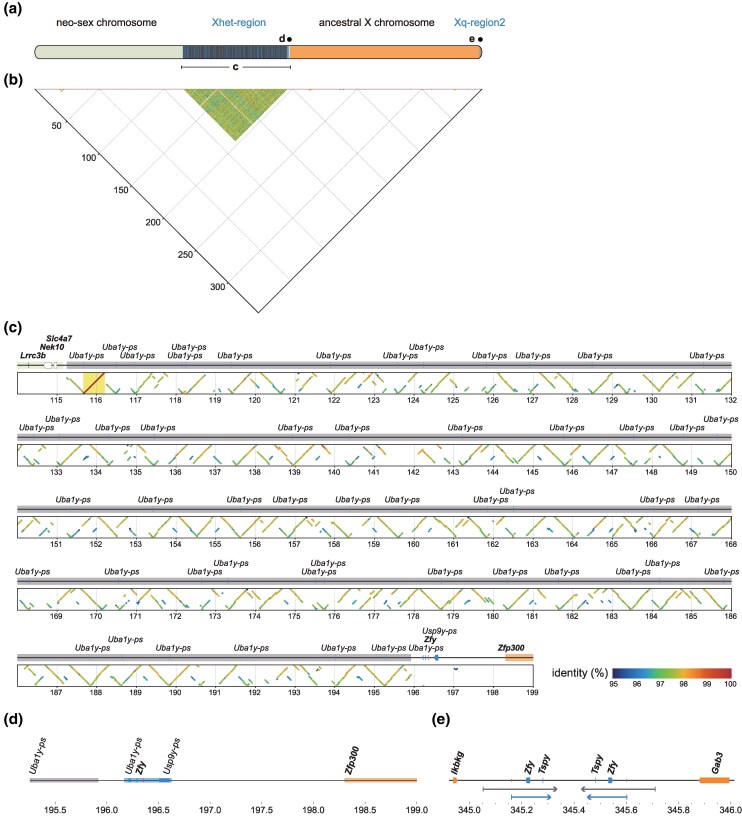
*Tokudaia* Y-MRCA-derived region in X chromosome of *T. muenninki.* a) Overview of the *Tokudaia* Y-chromosomal most recent common ancestor (Y-MRCA)-derived region identified in the X chromosome of *T. muenninki*. This region appears in two distinct locations: the Xhet-region and Xq-region2. b) Self-dot-plot alignment of the neo-ancX chromosome of *T. muenninki*. The central region of neo-ancX consists of a heterochromatic region composed of repetitive elements. c) Enlarged view of the Xhet-region (115.2 to 198.3 Mbp). Genes and BASD are shown as horizontal lines in the genome. Regions of Y-chromosome origin, X-chromosome origin, heterochromatin, and BASD are distinguished by their labels and positions. We identified 52 copies of partial *Uba1y* (pseudogene) within the heterochromatic region spanning approximately 80 Mbp. On the 3′ side of this region, intact *Zfy*, pseudogenes of *Uba1y* and *Usp9y*, and BASD, are located. The ancX region begins at *Zfp300*. At the bottom, a dot-plot alignment diagram shows how the highlighted 542 kbp basic repeat unit aligns within the region. The heterochromatic region is composed of irregular repeats of a basic unit that includes partial *Uba1y*. Genes labeled with “-ps” represent pseudogenes, while intact genes are indicated in bold. d) Enlarged view of the 3′ side of the Xhet-region (195.3 to 199.0 Mbp). This figure shows an enlargement of the area between the heterochromatic region and the start of the ancX region, as shown in b). Within the genomic region depicted by boxed outlines, *Uba1y* pseudogenes, intact *Zfy*, BASD, and *Usp9y* pseudogenes are aligned. Genes labeled with “-ps” represent pseudogenes, while intact genes are indicated in bold. e) Enlarged view of Xq-region2 (344.9 to 346.0 Mbp). Between the X-linked genes *Ikbkg* and *Gab3*, a 162 kbp *T. muenninki*-specific insertion of the *Tokudaia* Y-MRCA-originated sequence, containing intact *Zfy*, *Tspy*, and BASD, was identified (indicated by the lower arrow). This sequence, along with an approximately 300 kbp region containing it (indicated by the upper arrow), has undergone an inverted duplication. Genes labeled with “-ps” represent pseudogenes, while intact genes are indicated in bold.

**Fig. 5.**
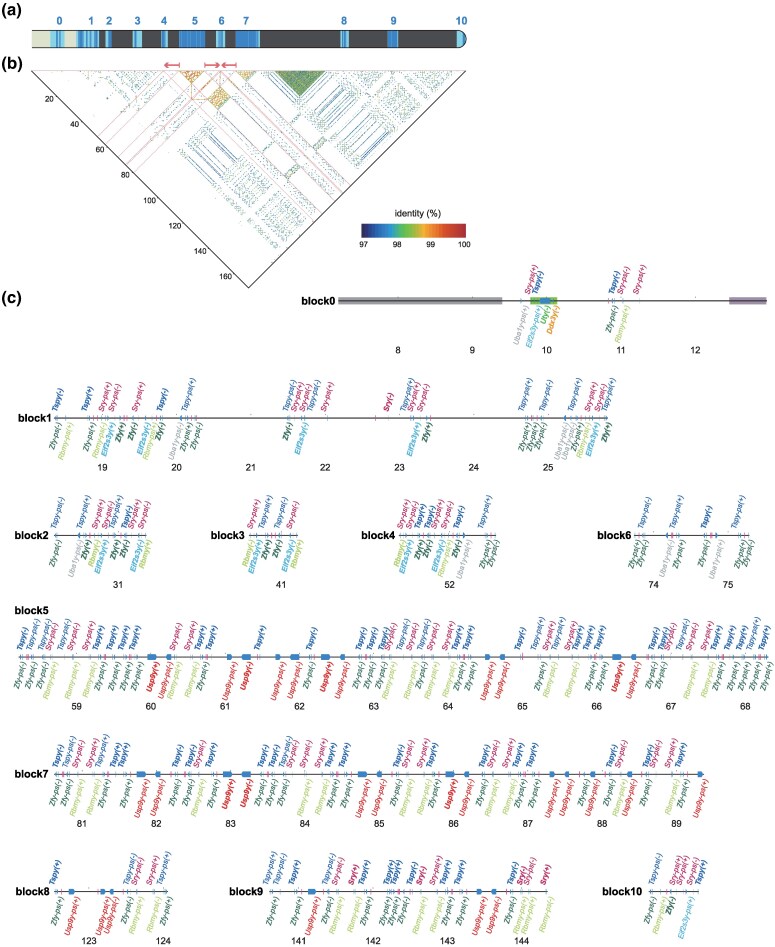
*Tokudaia* Y-MRCA-derived region in the Y chromosome of *T. muenninki.* a) Overview of the *Tokudaia* Y-chromosomal most recent common ancestor (Y-MRCA)-derived region identified in the Y chromosome of *T. muenninki*. The *Tokudaia* Y-MRCA-derived region spans a broad ancY-het-region on the Y chromosome. In the ancY-het-region, the ancY sequence is divided into blocks 1 to 10 by the heterochromatic regions, whereas block 0 exists on the proximal side of the neo-Y region. Blocks 0 and 1 are separated by the neo-Y-region-derived sequences. b) Self-dot-plot alignment of the Y chromosome of *T. muenninki*. The Y chromosome includes the genomic region extending from the differentiated neo-Y region to the distal end of ancY, spanning upstream approximately 100 Mbp into the neo-sex chromosome, with minimal differences compared to the neo-X chromosome. The neo-Y and ancY regions are closely adjacent. The highly duplicated *Tokudaia* Y-MRCA-originated region is divided into ten blocks by heterochromatic regions composed of repetitive sequences, forming a mosaic structure between the two regions. Arrows at the top of the figure indicate the repeat units (three copies each) that constitute the palindromic structures. c) Enlarged view of *Tokudaia* Y-MRCA-derived region blocks in the ancY region. The enlarged view of blocks 0 to 10 displays the Y-linked genes and BASD present in each block. Identical genes are shown in matching labels, corresponding to those listed in supplementary table S3, Supplementary Material online. The 372 kbp region previously identified via bacterial artificial chromosome-based analysis is highlighted in the figure. Genes labeled with “-ps” represent pseudogenes, while intact genes are indicated in bold.

Before discussing individual loci, we provide an overview of the sex chromosomes of *T. muenninki* based on our genome assembly. As shown in Figs. 4a and 5a, each of the ancestral X (ancX) and ancestral Y (ancY) chromosomes fused with one of the two homologous copies of the same autosome (forming neo-sex chromosomes), resulting in large, extended sex chromosomes. In this study, the terms “neo-X”, “neo-Y”, and “neo” are used in both structural and sequence contexts. Structurally, “neo-X” and “neo-Y” refer to the autosomal segments that have fused with the ancestral X and Y chromosomes, respectively, while “neo” refers more generally to the autosome-derived portion of the sex chromosomes. In sequence-based analyses, we use “neo-X” or “neo-Y” when sequence differences allow us to distinguish their origin, and “neo” when such differences are not evident. The X chromosome comprises an autosomal and ancX region, separated by an approximately 80 Mbp heterochromatic region consisting of repetitive sequences. Meanwhile, the Y chromosome consists of autosomal and ancY regions that are positioned in close proximity, unlike the X chromosome, where the corresponding regions are separated by a large heterochromatic region. Additionally, part of the *Tokudaia* Y-MRCA-originated region is located on the proximal side of the autosomal region. The ancY region alternates between highly repetitive heterochromatic sequences and Y-linked gene regions, forming a mosaic structure. The assembled Y chromosome sequence extends from the differentiated neo-Y region to the distal end of ancY. Details of the *Tokudaia* Y-MRCA-originated locus in the *T. muenninki* genome are discussed below.

The X chromosome contains Y-linked genes in two regions: the central Xhet-region and the distal Xq-region2. The Xhet-region (115.2 to 198.3 Mbp) primarily consists of heterochromatic sequences, with a distinct 2.5 Mbp sequence on the 3′ side. Approximately 76% of this heterochromatic region is composed of irregular repeats of a 542 kbp basic unit, with 52 partial copies of *Uba1y* at the ends (Fig. 4b and supplementary fig. S10a to e, Supplementary Material online). The following 2.5 Mbp region contains intact *Zfy*, pseudogenes of *Uba1y* and *Usp9y*, and BASD (Fig. 4c). In Xq-region2, a *T. muenninki*-specific *Tokudaia* Y-MRCA-derived 162 kbp insertion, containing intact *Zfy*, *Tspy*, and BASD, was identified. This sequence, along with an approximately 300 kbp region containing it, underwent inverted duplication (Fig. 4d and supplementary fig. S10f and g, Supplementary Material online).

The ancY region of the Y chromosome exhibits an uneven distribution of Y-linked genes, which are organized into ten distinct blocks (blocks 1 to 10; Fig. 5, supplementary figs. S11a and S12 and supplementary table S3, Supplementary Material online). Notably, the longest inter-block region lies between blocks 7 and 8, where highly repetitive ∼120 kbp ancY-specific sequences are densely present (supplementary fig. S11b and c, Supplementary Material online). These sequences are also found between other inter-blocks, with a total of 481 copies spanning 36.4 Mbp—accounting for approximately 23% of the entire ancY region (supplementary fig. S11d to f, Supplementary Material online). Additionally, the regions spanning blocks 4–5, 5–6, and 6–7—including parts of the adjacent gene-containing regions—form relatively long palindromic structures totaling approximately 6 Mb (Fig. 5b and supplementary table S4, Supplementary Material online). While large palindromes are a well-known feature of human and mouse Y chromosomes (Shirleen et al. 2014; Rhie et al. 2023), neither these palindromes nor the abovementioned ∼120 kb repetitive sequences found in the heterochromatic clusters showed detectable sequence similarity to human or mouse Y chromosomes in BLASTN searches (>70% identity), suggesting that these elements are unique to *T. muenninki*.

Turning our attention to the gene content, in addition to the gene clusters located within blocks 1 to 10, we identified a distinct Y-linked gene cluster near the proximal end of the neo-Y chromosome, referred to as block 0. This block is separated from block 1 by neo-Y-derived sequences. Most blocks contain highly duplicated sequences with multiple intact or pseudogenized copies of Y-linked genes (supplementary table S3, Supplementary Material online). However, *Kdm5d* was detected only as a partial fragment (exons 11 to 17, 7 out of 26 exons) in the block 1, with a frameshift mutation in exon 14. No autosomal copy was identified. As our analysis includes only genes with at least 50% coverage, *Kdm5d* was considered absent in *T. muenninki*. Further details are provided in supplementary fig. S13, Supplementary Material online. Notably, blocks (1), 2, 3, and 4, as well as blocks 5, (6), 7, (8), and 9, form groups with similar Y-linked gene copy numbers and high genomic similarity. In contrast, block 0 contains only *Ddx3y* and *Uty*, including the 372 kbp sequence identified in a previous BAC-based study (Fig. 5c) (Murata et al. 2016). The Y-linked genes in block 0 are mostly single-copy and show little homology with other blocks, distinguishing them from the others.

**Fig. 6.**
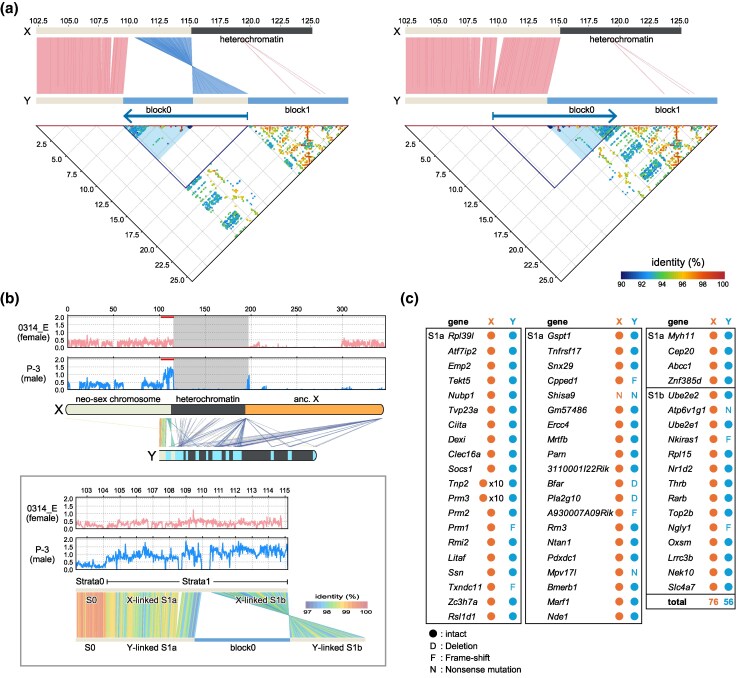
Comparative analysis of the block 0 surrounding region in the *T. muenninki* Y chromosome with the corresponding neo-X region. a) Comparative analysis of the *T. muenninki* Y chromosome blocks 0 to 1 surrounding region with the corresponding X chromosome region. The left panel shows a comparison of the current sequences. Upstream of block 0, a clear alignment between the neo-X and neo-Y regions is observed, while downstream, the neo-X region aligns with the Y chromosome region between block 0 and block 1 in an inverted orientation. Further downstream, the X chromosome forms heterochromatin, while the Y chromosome forms block 1. A self-dot-plot alignment of the corresponding Y chromosome region is shown at the bottom of the figure. In the right panel, the region from block 0 to just before block 1 (indicated by an arrow on the Y chromosome) is inverted. This operation connects the neo-X versus. neo-Y alignment directly up to the start of block 0, with the X chromosome forming heterochromatin and the Y chromosome displaying a continuous connection from block 0 to block 1. The dot-plot further confirms the continuity of repetitive sequences at the 5′ ends of both block 0 and block 1, forming a continuous repetitive sequence region. b) Distribution of heterozygous single nucleotide polymorphism (hetero-SNP) density across the entire X chromosome, excluding heterochromatin regions, along with a zoomed view of the 12.5 Mbp region surrounding the proximal end of the neo-sex chromosome. Data from the female individual are shown in the upper track, while data from the male individual are shown in the lower track. Below this expanded view, an alignment between neo-X versus. neo-Y (male) and neo-X versus. neo-X (female) are shown. Around the proximal end of the neo-sex chromosome (near 104.2 Mbp), the hetero-SNP density in the male sharply increases, while the hetero-SNP density in the female remains consistently low. Concurrently, the similarity between neo-X and neo-Y decreases, indicating areas of discontinuous differentiation. These areas were designated as Strata 0 (spanning from the distal end of the neo-sex chromosome to 104.2 Mbp) and Strata 1. While Strata 1 is further divided into two subregions (S1a and S1b) by block 0, no clear differences in hetero-SNP density were observed between them in the male. However, there was a slight tendency for higher hetero-SNP values in the male and lower neo-X versus. neo-Y identity near the heterochromatin. c) List of genes located in Strata 1 (neo-X and neo-Y regions). Tandem duplications of *Tnp2* and *Prm3* were observed on the neo-X side, whereas *Bfar* and *Pla2g10* were deleted on the neo-Y side. Additionally, eight genes were pseudogenized on the neo-Y side, indicating degeneration within the Y side of the neo region.

We then look into the region surrounding block 0. This region (7.2 to 12.9 Mbp) contains repetitive sequences at both the 5′ (7.2 to 9.4 Mbp) and the 3′ (12.5 to 12.9 Mbp) ends (Fig. 6a). Notably, just downstream of the 3′ repetitive sequences lies a neo-Y-chromosome-derived 4.6 Mbp sequence, which continues into block 1. Alignment with the X chromosome (Fig. 6a, left) revealed that this 4.6 Mbp neo-Y sequence between blocks 0 and 1 underwent an inversion relative to the homologous neo-X sequence. Synteny with homologous regions in *T. osimensis* and *T. tokunoshimensis* suggests that the inversion occurred on the Y chromosome (supplementary fig. S14, Supplementary Material online). When the neo-Y region (7.2 to 17.5 Mbp) is inverted, the neo-Y-chromosome-derived region becomes continuous (Fig. 6a, right), aligning the repetitive sequences at the 5′ end of block 0 with those at the 5′ end of block 1 to form a continuous block 0–1 region. This suggests that the neo-Y region originally had the genomic structure shown in Fig. 6a (right) and later underwent inversion to form the current structure (Fig. 6a, left).

In the male genome, heterozygous single nucleotide polymorphism (SNP) density and sequence identity between homologous chromosomes were examined across the X chromosome (Fig. 6b, blue line). A pronounced increase in heterozygous SNPs, along with a decrease in sequence identity, emerged near 104.2 Mbp, between *Grin2a* and *Rpl39l*, indicating the onset of sequence differentiation between the neo-X and neo-Y regions. In contrast, analysis of SNP density within a female individual (between two X chromosomes) (Fig. 6b, pink line) revealed uniformly low heterozygous SNP rates across the entire chromosome including neo-X region. These results suggest that recombination occurs between the neo-X and neo-Y regions from the distal end to 104.2 Mbp, defining this segment as the pseudoautosomal region (PAR, Strata 0), whereas the region beyond this point has undergone differentiation and no longer recombines. The differentiated region beyond 104.2 Mbp is referred to as Strata 1, which is further divided into two subregions the inversion described above into two subregions—Strata 1a (S1a) and Strata 1b (S1b)—by the inversion described earlier (Fig. 6b). However, no marked differences were observed between these two regions in terms of heterozygous SNP density or sequence divergence, suggesting that the inversion itself had limited impact on the overall degree of differentiation within Strata 1. Information on genes located in Strata 1 is presented in Fig. 6c. On the neo-Y side, eight genes have become pseudogenes with partial deletions, illustrating the ongoing degeneration of this region.

### Analysis of BASD

In the previous sections, we examined the current state of *Tokudaia* Y-MRCA-originated genomic regions, identifying not only Y-linked genes but also BASDs as significant elements within these regions. To further characterize BASD, we conducted homology searches against known sequence databases. When we performed a nucleotide search of identified BASDs, as well as a translated search (ORF-based) against the nonredundant databases, we did not find any homologous sequences outside rodent genomes. Additionally, no significant matches to known repetitive elements were detected, suggesting that BASD is a rodent-specific sequence. Based on this result, we expanded our investigation into the genomes of nine rodent species to determine the distribution of BASDs beyond the *Tokudaia* species. The results showed that, except for *Tokudaia* species, 1 to 2 copies of BASD were found only in regions corresponding to the Xq-region1 of other rodent genomes, with no copies present in other chromosomal regions (supplementary fig. S15, Supplementary Material online). In *T. osimensis* and *T. tokunoshimensis*, BASD was also observed exclusively in Xq-region1, with three and four copies, respectively, as noted earlier (Fig. 3a). Conversely, in *T. muenninki*, the BASD was found not only at two locations within Xq-region1 but also in two partial sequences within Xq-region2 (Fig. 4d), one in the terminal ancX region of the Xhet-region (Fig. 4c), one in the Y chromosome block 0 region, and at 104 locations across blocks 1 to 10 (Fig. 5b, supplementary tables S3 and S5, and fig. S16, Supplementary Material online). This suggests that, while BASD is typically restricted to two sites within Xq-region1 across rodents, it has undergone duplication and translocation to regions harboring Y genes in *Tokudaia* species, particularly in *T. muenninki*, where it has extended beyond Xq-region1.

**Fig. 7.**
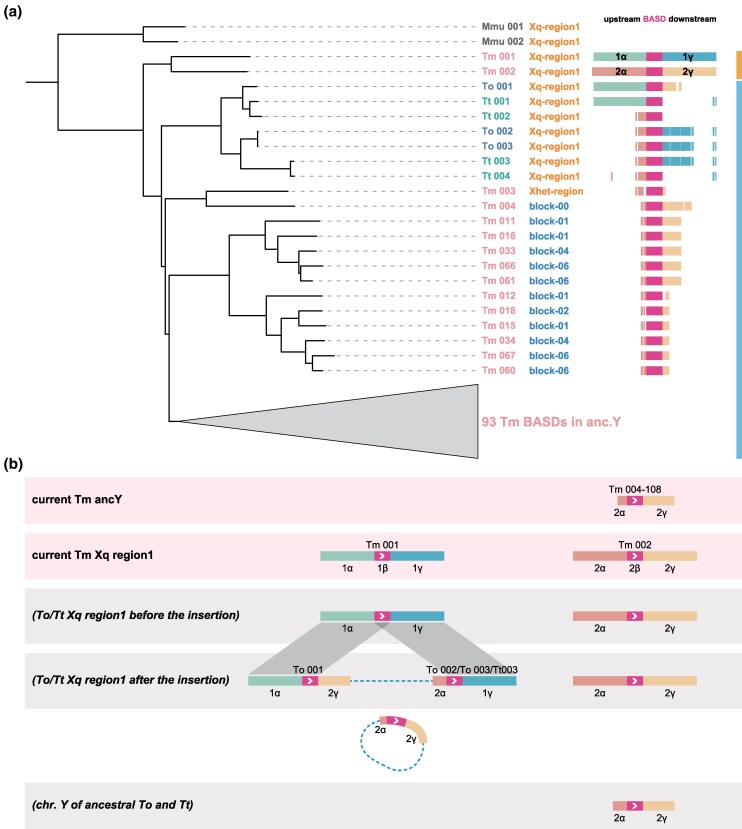
Phylogenetic relationships of the boundary-associated segmental duplications (BASDs) in *Tokudaia* and mouse genomes, with alignment to *T. muenninki*'s ancestral BASDs (Tm-BASD001 and Tm-BASD002). a) Left: Phylogenetic tree showing the relationship among BASDs found in the genomes of the three *Tokudaia* species and mouse. Right: Regions around each BASD that align with the flanking sequences of the putative ancestral BASDs are distinguished based on their correspndence to Tm-BASD001 and Tm-BASD002. Among the BASDs identified in the ancY region, 93 were collapsed for clarity. The full tree with the expanded clade is available in supplementary fig. S17, Supplementary Material online. b) Based on the results shown in a), this panel illustrates the current configuration of sequences flanking each BASD, as well as the presumed ancestral state surrounding BASD001 prior to the insertion of *Tokudaia* Y-MRCA-derived sequences into the Xq-region1 in *T. osimensis* and *T. tokunoshimensis*. Dashed lines indicate *Tokudaia* Y-MRCA-derived segments. The close proximity of α, BASD, and γ supports a model in which these sequences formed a circular intermediate (eccDNA) prior to integration.

To further clarify how the BASD and its surrounding regions are distributed within the *Tokudaia* genus, we analyzed the evolutionary relationships based on BASD homology. Figure 7 and supplementary fig. S17, Supplementary Material online illustrate the phylogenetic tree of the identified BASDs, along with the alignment results for each BASD-surrounding region, against the two BASD-flanking sequences in *T. muenninki*, which are considered to retain the ancestral configuration. The tree shows a clade consisting first of mouse BASDs, followed by BASDs from *T. muenninki*'s Xq-region1 (indicated by the vertical orange bar). Below this, BASDs found outside Xq-region1 in *T. muenninki*, as well as those found in Xq-region1 of *T. osimensis* and *T. tokunoshimensis*, form a large single clade, all of which are considered to be contained within the *Tokudaia* Y-MRCA-derived regions (indicated by the vertical blue bar). Additionally, a comparison with the ancestral-type Tm-BASD-flanking regions revealed that these BASDs aligned with much of the surrounding region of Tm-BASD002 (up to 1.8 kbp upstream [2α] and 10.8 kbp downstream [2γ]), suggesting that these sequences likely originated from a translocation of the ancestral Tm-BASD002-flanking region on the X chromosome to the Y chromosome (X-to-Y event), with no discrepancies. Furthermore, the upstream region of Tm-BASD001 corresponds to To-BASD001 and Tt-BASD001, while its downstream region aligns with To-BASD002, To-BASD003, and Tt-BASD003, confirming that the *Tokudaia* Y-MRCA-derived sequence was inserted between SB1 and SB2 in both *T. osimensis* and *T. tokunoshimensis*.

Further analysis of the downstream region of To-BASD001 revealed that it corresponds to the downstream region of Tm-BASD002 (2γ), while the upstream region of To-BASD002 corresponds to the upstream region of Tm-BASD002 (2α; Fig. 7b). This suggests that the inserted sequence between SB1 and SB2 in *T. osimensis* and *T. tokunoshimensis* has a structure of 2γ-[Y and autosome-derived sequence]-2α, with BASD sequences positioned at both insertion boundaries. Given that the original arrangement of these sequences was 2α-BASD-2γ, the question arises as to why they are now separated into BASD-2γ and 2α-BASD flanking the inserted sequence. One plausible explanation is that the 2α-BASD-2γ-[Y and autosome-derived sequences] initially formed an extrachromosomal circular DNA (eccDNA) (Zhao et al. 2022; Holt et al. 2023) intermediate, which subsequently integrated into the X chromosome via NAHR mediated by the BASD sequence.

## Discussion

The *Tokudaia* genus represents a remarkable exception to the conserved XX/XY sex chromosome system prevalent in mammals. Through our chromosome-level genome assemblies and comparative analyses, we conducted a comprehensive exploration of the current *Tokudaia* Y-MRCA-derived regions in all three species and identified BASDs as key structural elements specifically present in these regions. Furthermore, by elucidating the evolutionary traces remaining in the genome mediated by BASDs, we shed light on the evolutionary trajectory of *Tokudaia* Y-MRCA-derived regions.

**Fig. 8.**
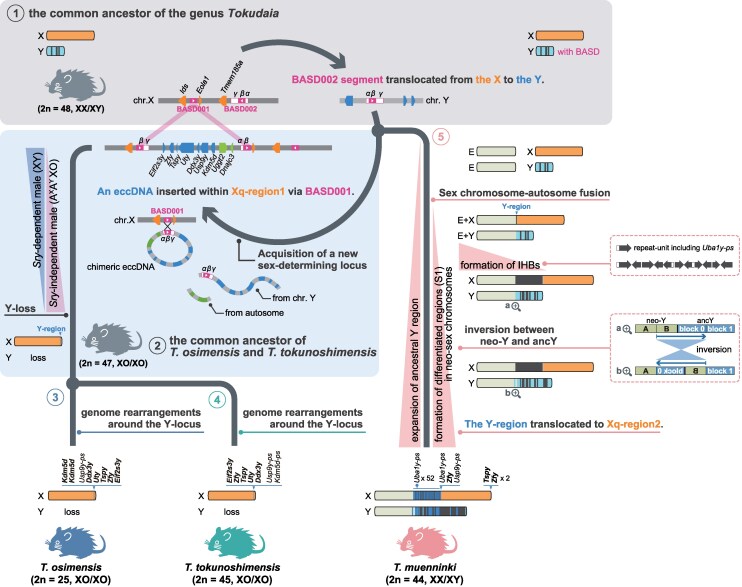
Schematic diagram of Y chromosome-related evolutionary events inferred for the three *Tokudaia* species based on the results of previous analyses.

Based on these results, we propose the most plausible scenario for the evolution of *Tokudaia* Y-MRCA regions, which involves multiple translocation events, as summarized in Fig. 8. Initially, in the common ancestor of the genus *Tokudaia*, the BASD002 and its surrounding regions (α-β-γ, where β represents the BASD itself) from Xq-region1 were duplicated and translocated to *Tokudaia* Y-MRCA (Fig. 8-(1), X-to-Y event). After the divergence of *T. muenninki*, the SB6–SB5 region (*Tokudaia* Y-MRCA-originated region with an autosome-derived segment), including BASD, formed eccDNA in the common ancestor of *T. osimensis* and *T. tokunoshimensis*. This induced NAHR with BASD001, resulting in the insertion of *Tokudaia* Y-MRCA-originated sequences into the X chromosome and the replacement of the ancestral BASD001 with Y-derived BASD (Fig. 8-(2)). Subsequently, inversions and duplications formed SB5′, and after the divergence of *T. osimensis* and *T. tokunoshimensis*, further inversions led to their current genome structures (Fig. 8-(3) and (4)). The present hypothesis suggests two translocations: one from the X chromosome to the Y chromosome and the other from Y to X, a phenomenon similar to the formation of the cattle color-sidedness locus through two translocations via circular intermediates (Durkin et al. 2012). Additionally, it has been shown that the insertion sequence into the X chromosome contains not only Y-derived sequences but also fragments of autosomal origin. Given that 20% of eccDNA found in human germline cells is composed of multiple genomic segments (Henriksen et al. 2022), it is plausible that autosomal and Y-derived segments formed chimeric structures during eccDNA formation, subsequently co-integrated into the X chromosome.

In *T. muenninki* (Fig. 8-(5)), autosomes fused with the X and Y chromosomes, already containing duplicated BASDs, forming sex chromosomes with large pseudoautosomal regions. The ancY-het-region contains Y-linked genes, such as *Ddx3y* and *Uty*, which are only found in block 0, suggesting that block 0 retains the ancestral state. Furthermore, as shown in the phylogenetic tree of BASDs (Fig. 7a), the BASD located in block 0 diverges earliest among all BASDs identified in the *Tokudaia* Y-MRCA-derived regions of *T. muenninki*. This strongly suggests that block 0 represents the initial site of BASD insertion during the earliest X-to-Y translocation event (Fig. 8-(1)).

Successive duplications of the ancestral Y regions likely formed blocks 1 to 10, while recombination suppression at the proximal end of the neo-sex chromosomes fostered the development of divergent regions. An inversion involving block 0 and the terminal neo-Y region may have contributed to the current neo-Y–ancY structure. The presence of multiple BASD-*Zfy-Tspy* sets in the Xq-region2 and ancY regions suggests that a portion of the Y chromosome translocated into Xq-region2.

The Xhet-region (the heterochromatic region between the neo-X chromosome and the ancestral X chromosome) likely serves as an intercalary heterochromatic block (IHB), preventing Xist-driven gene dosage compensation from affecting neo-X regions. IHBs are thought to form early after autosome-sex chromosome fusion. While IHBs have been reported in other rodents (Dobigny et al. 2004; Veyrunes et al. 2004; Deuve et al. 2006; Oliveira da Silva et al. 2022), specific IHB sequences were first identified in *T. muenninki*. Partial *Uba1y* copies were found within the repetitive elements comprising the Xhet-region, with Y-linked sequences, including BASD, located at the last 2.5 Mbp of the Xhet-region. These sequences may have been acquired through translocations between the X and Y chromosomes, with duplications forming the current IHBs spanning approximately 80 Mbp (Fig. 4c).

To further understand the integration of *Tokudaia* Y-MRCA-derived sequences into Xq-region1 and Xq-region2, we examined both the genes incorporated and the genomic regions where these sequences were inserted. Although *T. osimensis* and *T. tokunoshimensis* have lost their Y chromosomes, some genes originally encoded in Xq-region1, such as *Zfy*, *Eif2s3y*, *Uty*, *Tspy*, and *Ddx3y*, remained intact. These genes may have been preserved primarily due to their essential role in male development, as suggested by previous studies on Y-linked gene conservation across mammals (Bellott et al. 2014). The survival of dosage-sensitive genes on the Y chromosome has been linked to their function in transcriptional and translational regulation, highlighting their significance beyond male-specific roles (Bellott et al. 2014). The retention of Y-linked genes has also been observed in other rodents that have lost their Y chromosome. A comparative analysis of Y-linked genes across *Ellobius* species (Mulugeta et al. 2016), *Microtus oregoni* (Couger et al. 2021), and *Tokudaia* (this study) is summarized in Table 2. This comparison highlights that *Eif2s3y* is consistently retained in all examined species that have lost their Y chromosome, reinforcing its essential role in male fertility. Prior studies have shown that *Eif2s3y* is sufficient to drive spermatogonial differentiation, enabling the formation of haploid germ cells that are functional in assisted reproduction, even in the absence of other Y-linked genes (Yamauchi et al. 2014). Additionally, *Zfy* is retained in most species, although it has lost some exons in *M. oregoni*. This supports previous findings that highlight the importance of *Zfy* in spermatogenesis (Yamauchi et al. 2022). Interestingly, some of these genes, including *Eif2s3y*, are also present in the X chromosome (X^M^), which is shared by both sexes of *M. oregoni* (Table 2). Although their retention in females may be selectively neutral, current evidence strongly supports their essential role in male viability. After the divergence of the two *Tokudaia* species, duplication and inversion events were observed; however, the synteny of the seven Y-linked genes on SB5 was maintained, further supporting strong selective pressure on the X chromosome.

We next examined the genomic context of the insertion sites. The presence of BASD is a key factor in these regions. Structural rearrangements observed in both Xq-region1 and Xq-region2 across various species suggest that these regions are susceptible to structural variation and may have been receptive to the insertion of *Tokudaia* Y-MRCA-derived sequences (supplementary figs. S10f and S18, Supplementary Material online). Instability in the syntenic regions of Xq-region1 has also been observed in mice and humans (Warburton et al. 2004; Mueller et al. 2008; Swanepoel et al. 2020; Jackson et al. 2021), supporting the idea that structural flexibility facilitated rearrangements. While large segmental duplications are present in the Xq-region1 of humans, mice, and other species, they are spatially separated from BASD and exhibit lineage-specific variation in structure and gene content (supplementary fig. S18, Supplementary Material online), indicating that this region is structurally unstable and prone to recurrent rearrangements.

Despite these advances—namely, the identification of the initial X-to-Y translocation and the subsequent incorporation of *Tokudaia* Y-MRCA-derived regions into structurally dynamic areas of the X chromosome mediated by BASD—key questions remain. Chief among them is the functional role of BASD and its contribution to the structural stability of these chromosomes, as well as the precise molecular mechanisms driving the observed translocations. While our study focuses on elucidating how these chromosomal rearrangements have occurred, the ultimate evolutionary pressures that led to these changes remain an open question. Investigating the ecological, reproductive, and genetic factors influencing Y chromosome loss in *Tokudaia* will be crucial for future studies. Another major question is the novel sex determination system that arose in *T. osimensis* and *T. tokunoshimensis*. While we have characterized the genomic changes associated with this transition, the regulatory mechanisms governing male differentiation remain unclear. Investigating how these species compensate for the loss of the Y chromosome is essential for understanding the broader evolutionary forces driving sex chromosome transformation.

Previous studies on the creeping vole (*M. oregoni*) and *Ellobius* have provided key insights into the retention of Y-linked genes, but have primarily focused on gene-level analyses rather than large-scale chromosomal architecture. Structural analyses of genome reorganization following Y chromosome loss remain largely unexplored in these species. In contrast, our study leveraged high-quality, chromosome-level genome assemblies of three closely related *Tokudaia* species, enabling a detailed characterization of the structural rearrangements accompanying Y chromosome loss. Particularly, the ability to compare *T. osimensis* and *T. tokunoshimensis*, which diverged only ∼1 million years ago, has provided an unprecedented opportunity to track lineage-specific chromosomal changes within Y-derived regions. Further challenges include the difficulty of functional validation due to the endangered status of *Tokudaia* and the lack of an outgroup species with a typical XX/XY system, particularly one with a fully assembled Y chromosome genome. Addressing these gaps will require innovative approaches, such as structural modeling and comparative analyses with closely related rodent species.

In conclusion, this study provides a foundational genomic resource and a crucial step toward understanding the mechanisms and evolutionary implications of Y chromosome loss. By integrating detailed structural and comparative analyses, we establish *Tokudaia* as a key model for investigating rapid sex chromosome evolution and underscore the importance of further comparative studies to fully elucidate the genomic consequences of Y chromosome loss in mammals.

## Materials and Methods

### Genome Reconstruction of *T. muenninki* Using PacBio HiFi Reads

The whole genome assembly of *T. muenninki* was reconstructed by newly obtaining PacBio HiFi reads and assembling them together with previous data. The remaining DNA obtained during the analysis in a previous study was used for sequencing. Genomic DNA was sheared into fragment sizes ranging from 15 to 20 kb using the Megaruptor 3 system (Hologic, MA, USA). A HiFi library was prepared using a SMRTbell Prep Kit 3.0 (Pacific Biosciences, CA, USA), followed by size selection using the Blue Pippin system (Sage Science, MA, USA) to remove short fragments. The library was sequenced on the Revio system using a Revio polymerase kit (Pacific Biosciences, CA, USA) and a Revio sequencing plate (Pacific Biosciences, CA, USA) with 30-h movies per SMRT cell. A total of 201.5 Gb of HiFi reads were generated from two Revio SMRT cells and processed using the PacBio SMRT Link v13.1.0.221972 software.

In addition to the newly obtained HiFi reads, previously acquired PacBio CLR reads and Arima Hi-C reads from the same individual were used as input. Assembly was performed using Hifiasm v0.19.8 (Cheng et al. 2021), treating the PacBio CLR reads as ultra-long reads. Following this, Hi-C scaffolding was applied to achieve chromosome-level assembly. Examination of the input assembly contigs prior to Hi-C scaffolding revealed the presence of sex chromosome-derived contigs that were not included in the p_ctg output of the Hifiasm assembly. Consequently, these sex chromosome-derived contigs—identified in hap1_ctg and hap2_ctg based on Hi-C contact information—were reverted, and Hi-C scaffolding was then conducted using YaHS v1.2 (Zhou et al. 2023) with the Arima Hi-C reads. The Hi-C contact map was visualized using Juicebox v1.11.0828 (Robinson et al. 2018), and extensive manual curation was performed to fix mis-assemblies and mis-scaffoldings. Supplementary fig. S11, Supplementary Material online illustrates the Hi-C contact map constructed from the final sequences.

### Chromosome-to-Chromosome Alignment and Prediction of Karyotype Evolution

For pairwise sequence alignments between the three *Tokudaia* species, the query sequences were first fragmented into 500 kbp segments using the “seqkit” sliding command (Shen et al. 2016) and aligned to the reference genome using minimap2 v2.23 with the -c option (Li 2018). Only primary alignments exceeding 100 kbp were visualized using a lyla-plot. Genomic alignments enabled the prediction of chromosome fusion events in each of the three species, as along with the karyotype of the common ancestors of *T. osimensis* and *T. tokunoshimensis* and the genus *Tokudaia*. The karyotypes of these species and their ancestors were visualized on a unified scale using the RIdeogram library in the R package (Hao et al. 2020).

### Prediction of Y-Linked Genes

We identified Y-linked genes across the entire genome of *T. osimensis* by manually integrating homology- and RNA-seq-based predictions. For homology-based predictions, *M. musculus* protein sequences and previously reported *T. osimensis* proteins were splice-aligned to the genome using Spaln v2.3.3 (Iwata and Gotoh 2012) to predict gene structures. For RNA-seq-based predictions, RNA-seq reads from male and female brains (SRR8429976 to SRR8429982) were mapped to the genome using HISAT2 v. 2.2.1 (Kim et al. 2019), and the mapped reads were assembled into candidate transcripts using StringTie v2.2.0 (Kovaka et al. 2019). The open reading frames (ORFs) of the predicted transcripts were identified using TransDecoder (https://github.com/TransDecoder/TransDecoder). For *T. tokunoshimensis*, Y-linked gene structures were predicted by splice-aligning *T. osimensis* Y-linked genes using Spaln v2.3.3. To predict multicopy Y-linked genes from the *T. muenninki* genome, comprehensive gene structure prediction was conducted in two steps: (i) identification of homologous loci for the Y-linked genes and (ii) prediction of the exon–intron structure of the Y genes at each locus. First, the Y-linked genes of *T. osimensis* (or *M. musculus* when *T. osimensis* data were unavailable) were aligned to the genome using TBLASTN (Altschul et al. 1990). Regions extending 10 kbp upstream and downstream of the alignment segments, covering more than 50% of the query length, were extracted. For each locus, the exon–intron structure was predicted on the chromosome masked outside the homologous Y-gene region using Exonerate (Slater and Birney 2005). Finally, genes with the longest ORFs, covering at least 80% of the query length, were classified as intact, whereas others were considered pseudogenes.

### Prediction of Genome Rearrangements in Y-Originated Loci of *T. osimensis* and *T. tokunoshimensis* Using Maximum Parsimony

We aimed to predict the insertion loci of the Y-derived sequence in the common ancestor of *T. osimensis* and *T. tokunoshimensis*, as well as the inversion events that occurred in their respective lineages. First, SBs were manually defined for the three *Tokudaia* species and mouse genomes, based on interspecies alignments using minimap2 v2-2.24 and MAFFT v7.490 (Katoh and Standley 2013). We then inferred the order and orientation of the SBs in the common ancestor of the two species using the maximum parsimony method, making the following three assumptions: (i) the order and orientation of the SBs before the insertion of the Y-derived sequence into the X chromosome of the common ancestor was SB1(+)–SB2(+)–SB3(+)–SB4(+); (ii) SB5 and SB6, originating from the Y chromosome, were inserted together at the same locus; and (iii) duplicated sequences, including SB5′, were excluded when calculating the edit distance. We considered three candidate insertion loci (between SB1 and SB2, between SB2 and SB3, and between SB3 and SB4) and eight possible combinations of the orientation and order of SB5 and SB6 as potential inserted sequence structures in the common ancestor of the two species. For these 24 combinations, we calculated the minimum number of inversions required to derive the current structure of *T. osimensis* and *T. tokunoshimensis* from the ancestral structure, calculated separately for each species using UniMoG (Hilker et al. 2012).

### Homologous Search of BASDs and Phylogenetic Analysis

To identify BASDs, we aligned the genomic sequences of the mouse BASD (5,908 bp and 5,870 bp) as queries using BLASTN (Altschul et al. 1990). Alignments shorter than 500 bp were excluded, while split alignments within 3,000 bp of the genomic position were merged. Regions with alignments longer than 3,000 bp were considered potential BASDs. This process was repeated iteratively, using the candidate sequences as new queries. Sequences longer than 5,000 bp were designated as final BASDs for further analysis. Multiple sequence alignments of obtained BASDs were performed using MAFFT v7.4904, removing spurious sequences or poorly aligned regions with trimAI v1.4.16 (Capella-Gutiérrez et al. 2009). Phylogenetic analysis was performed using IQ-TREE v1.6.12 (-b 1000) (Nguyen et al. 2015) and visualized using iTOL (Letunic and Bork 2021).

### Detection of the Basic Repeating Unit in Heterochromatin Regions of *T. muenninki* Sex Chromosomes

To investigate the repetitive units within the heterochromatic region between the neo-sex chromosome and ancestral X on the X chromosome of *T. muenninki*, a self-alignment of the X chromosome from 114 to 120 Mbp was performed using BLASTN. This region encompasses the terminal portion of the neo-sex chromosome and a portion of the adjacent heterochromatin. The alignment results were visualized using a dot-plot diagram. According to the dot-plot diagram, the repetitive unit (115,656,799 to 116,199,009 bp) of the heterochromatin was manually identified. Subsequently, the region from 114 to 199 Mbp of the X chromosome was aligned to the repetitive unit using BLASTN, and the alignment results were visualized in dot-plot diagrams. Similarly, to investigate the repetitive units within the heterochromatic region of the Y chromosome, a self-alignment of the Y chromosome from 104.9 to 107.0 Mbp, corresponding to the heterochromatic region between blocks 7 and 8, was performed using BLASTN. The entire region from 90 to 122 Mbp of the Y chromosome was aligned to a manually determined repetitive unit (104,993,405 to 105,113,388 bp) using BLASTN, and the alignment results were visualized in dot-plot diagrams.

### Detection of Heterozygous SNPs on the Neo-Sex Chromosome of *T. muenninki*

Illumina reads were mapped to the *T. muenninki* genome, excluding the Y chromosome, using bwa-mem2 v2.2.1 (Vasimuddin et al. 2019). Reads with less than 90% identity or less than 80% alignment coverage were filtered out using the Samtools view command (Li et al. 2009). SNP calling was performed using Bcftools v1.13 (Danecek et al. 2021) with the “mpileup” command (parameter: -d 200), followed by the “call” command with the -m option. Heterozygous SNPs were selected based on the following criteria: single nucleotide substitutions, allele frequency (AF) between 0.25 and 0.75, and read depth between 21 (half of the mode) and 65 (1.5 times the mode).

### Gene Prediction of the Neo-Sex Chromosome S1 Region in *T. muenninki*

We predicted gene structures within the differentiation regions (Strata 1) of the neo-sex chromosomes by combining homology- and RNA-seq-based approaches. For homology-based prediction, the protein sequences from *M. musculus*, *Rattus rattus*, and previously annotated *T. muenninki* sequences were splice-aligned to the sex chromosomes using Spaln v2.3.3 to predict gene structures. For the RNA-seq-based prediction, RNA-seq reads from the male brain, liver, and testis (DRR059293 to DRR059295), as well as from male and female fibroblasts (DRR059296 to DRR059302) of *T. muenninki*, were mapped to the sex chromosomes using HISAT2 v. 2.2.1. The mapped reads were assembled into candidate transcripts using StringTie v2.2.0, and ORFs of the predicted transcripts were identified using TransDecoder.

## Supplementary Material

msaf102_Supplementary_Data

## Data Availability

The HiFi sequencing data for *T. muenninki* have been submitted to DDBJ under accession numbers DRR613121 and DRR613122. The genome assemblies for *T. muenninki* have been deposited in DDBJ under accession numbers BAAFZZ010000001 to BAAFZZ010000034.
