# Supplementary material for: Where Did the Y Chromosome in the Spiny Rat Go, and How Did It Get There?
Source: Mol Biol Evol. 2025 May 6;42(5):msaf102. doi: 10.1093/molbev/msaf102 (PMC12104558; doi:10.1093/molbev/msaf102)
Supplement: msaf102_Supplementary_Data [file msaf102_supplementary_data.pdf]

Supplementary methods

Phylogenetic analysis

We performed a phylogenetic analysis using twelve sets of protein sequences from the three *Tokudaia* species and nine rodent subfamily species. Only the longest isoform of each gene was used for the analysis. Single-copy orthologous genes were searched using SonicParanoid v1.3.5 (Cosentino and Iwasaki 2019). and 16,439 single-copy orthologous gene groups were identified. The amino acid sequences of each gene group were aligned using Mafft v7.481 (Katoh and Standley 2013) , and spurious sequences or low-quality aligned regions were trimmed using trimAl v1.4.1 (Capella-Gutiérrez et al. 2009) . The resulting amino acid sequence alignments for each gene were concatenated to construct a phylogenetic tree based on the maximum likelihood method using IQ-TREE v1.6.12 (Nguyen et al. 2015) . The phylogenetic tree was visualized using FigTree v1.4.4 (https://github.com/rambaut/figtree).

Chromosome-to-chromosome alignments between *T. muenninki* and *A. sylvaticus*

To confirm whether the two chromosome fusions occurred in the *T. muenninki* lineage, we performed chromosome comparisons using *A. sylvaticus*, the closest related species with a chromosome-level genome available, as an outgroup. For pairwise sequence alignments between *T. muenninki* and *A. sylvaticus*, the chromosome sequences of *A. sylvaticus* were first fragmented into 500 kbp segments using the seqkit sliding command (Shen et al. 2016) and aligned to the chromosome sequences of *T. muenninki* as the reference using minimap2 v2.23 with the -c option (Li 2018) . Only primary alignments with ≥ 100 kbp were visualized.

Comparison between *T. osimensis* Xq-region1 and the entire *T. muenninki* Y chromosome.

To search conserved synteny, we performed a BLASTN search (Altschul et al. 1990) between *T. osimensis* Xq-region1 (X chromosome: 135,674,126–136,923,198 bp) and the entire *T. muenninki* Y chromosome. The *T. osimensis* Xq-region1 sequence was used as the database after repeat masking while the *T. muenninki* Y chromosome was fragmented into 50 kbp segments using the seqkit sliding command and used as the query sequences. Alignments with ≥90% sequence identity and ≥1 kbp alignment length were visualized.

Supplementary tables

Supplementary Table 1. Positions of the synteny blocks (SBs) on the X chromosome in *Mus musculus* and the three *Tokudaia* species

species	Synteny Block	start position	end position	length (bp)
M. musculus	SB1	69,059,916	69,413,392	353,477
	SB2	69,419,301	69,612,350	193,050
	SB3	69,618,221	70,398,218	779,998
	SB4	70,398,219	70,707,398	309,180
T. muenninki	SB1	339,646,547	339,993,344	346,798
	SB2.1	339,999,686	340,037,867	38,182
	SB7	340,037,868	341,212,817	1,174,950
	SB2.2	341,212,818	341,319,711	106,894
	SB3	341,329,845	342,046,372	716,528
	SB4	342,046,373	342,354,357	307,985
T. osimensis	SB1	135,304,635	135,667,358	362,724
	SB6	135,674,126	135,723,967	49,842
	SB5	135,723,968	136,725,221	1,001,254
	SB2	136,731,562	136,824,014	92,453
	SB5'	136,830,355	136,923,197	92,843
	SB3	136,923,199	137,773,984	850,786
	SB6	137,782,222	138,089,393	307,172
T. tokunoshimensis	SB1	134,400,001	134,761,889	361,889
	SB3	134,778,653	135,569,457	790,805
	SB5'	135,569,458	135,623,990	54,533
	SB5	135,630,761	136,455,012	824,252
	SB2	136,466,454	136,580,404	113,951
	SB6	136,594,549	136,635,529	40,981
	SB4	136,693,160	137,030,336	337,177

Supplementary Table 2. Detailed information on the boundary-associated segmental duplications (BASDs) found in Xq-region1 of the three *Tokudaia* species and *Mus musculus*

species	chromosome	accession	prefix	start	end	strand	length (bp)
T. muenninki	X	neo-ancX	Tm-BASD001	339,993,344	339,999,684	+	6,341
	X	neo-ancX	Tm-BASD002	341,319,711	341,325,619	-	5,909
T. osimensis	X	Chr_scaffold_X	To-BASD001	135,667,357	135,674,125	+	6,769
	X	Chr_scaffold_X	To-BASD002	136,725,222	136,731,561	+	6,340
	X	Chr_scaffold_X	To-BASD003	136,824,015	136,830,354	-	6,340
T. tokunoshimensis	X	Chr_scaffold_X	Tt-BASD001	134,761,888	134,768,652	+	6,765
	X	Chr_scaffold_X	Tt-BASD002	135,623,991	135,630,760	+	6,770
	X	Chr_scaffold_X	Tt-BASD003	136,460,112	136,466,453	+	6,342
	X	Chr_scaffold_X	Tt-BASD004	136,674,587	136,680,929	-	6,343
M. musculus	X	NC_000086.8	Mm-BASD001	69,413,393	69,419,300	+	5,908
	X	NC_000086.8	Mm-BASD002	69,612,351	69,618,220	-	5,870

Supplementary Table 3. Number of Y-linked genes in each *Tokudaia* Y-MRCA derived blocks 0–10 in the ancY region of *T. muenninki*

block	*Ddx3y*	*Uty*	*Eif2s3y*	*Uba1y*	*Usp9y*	*Sry*	*Rbmy*	*Tspy*	*Zfy*	*Kdm5d*	BASD
0	1	1	1	1		3	1	2	1		1
1			5	3		11	4	11	14		13
2			2	1		4	2	4	4		4
3			2			2	2	2	2		6
4			2	1		4	2	4	5		7
5					12	14	13	33	25		[illegible]5
6				2				4	6		8
7					13	13	11	21	21		[illegible]5
8					3	2	2	3	3		5
9					3	7	7	11	10		10
10			1			3	1	2	2		1
total	1	1	13	8	31	63	45	97	93	0	105

Supplementary Table 4. Summary of palindromic structures identified in the *Tokudaia muenninki* Y chromosome and comparison with known palindromes in *Homo Sapiens* and *Mus musculus* Y chromosomes

species	start	end	length	name	direction	reference
M. musculus	10,111,975	14,762,205	4,650,231	4.5 Mbp tandem-repeats	+	Soh et al. 2014
	14,762,206	19,439,780	4,677,575	4.5 Mbp tandem-repeats	+	
	46,660,001	53,312,542	6,652,542	7Mbp direct repeats	+	
	59,125,620	65,773,712	6,648,093	7Mbp direct repeats	+	
H. sapiens	14,891,106	14,926,304	35,199	P8	−	Rhie et al.2023
	14,929,718	14,964,927	35,210	P8	+	
	16,781,335	16,790,043	8,709	P7	−	
	16,802,684	16,811,393	8,710	P7	+	
	17,066,094	17,176,113	110,020	P6	−	
	17,222,323	17,332,346	110,024	P6	+	
	18,362,331	18,857,802	495,472	P5	−	
	18,861,259	19,356,680	495,422	P5	+	
	19,356,701	19,546,840	190,140	P4	−	
	19,586,495	19,776,671	190,177	P4	+	
	22,760,852	23,044,266	283,415	P3	−	
	23,213,315	23,496,719	283,405	P3	+	
	23,897,358	24,035,399	138,042	P2	−	
	24,037,534	24,167,130	129,597	P2	+	
	24,167,431	25,503,792	1,336,362	P1	+	
	25,787,351	27,122,770	1,335,420	P1	+	
T. muenninki	52,142,760	58,374,919	6,232,160	large repeat	+	this study
	68,154,220	74,225,089	6,070,870	large repeat	−	
	74,793,549	80,780,818	5,987,270	large repeat	+	

Supplementary Table 5. Detailed information of the boundary-associated segmental duplications (BASDs) found outside Xq-region1 in *T. muenninki*

chromosome	region	prefix	start	end	strand	length (bp)
neo-ancX	Xhet-region	Tm_003	196,351,474	196,357,423	-	5,950
neo-Y-ancY	block0	Tm_004	10,826,432	10,832,356	-	5,925
neo-Y-ancY	block1	Tm_005	18,308,878	18,315,187	-	6,310
neo-Y-ancY	block1	Tm_006	18,876,003	18,882,341	+	6,339
neo-Y-ancY	block1	Tm_007	18,929,647	18,936,012	-	6,366
neo-Y-ancY	block1	Tm_008	19,289,454	19,295,761	+	6,308
neo-Y-ancY	block1	Tm_009	19,328,938	19,335,279	+	6,342
neo-Y-ancY	block1	Tm_010	19,725,641	19,731,934	-	6,294
neo-Y-ancY	block1	Tm_011	20,195,805	20,202,151	+	6,347
neo-Y-ancY	block1	Tm_012	20,263,138	20,269,487	-	6,350
neo-Y-ancY	block1	Tm_013	22,670,890	22,676,809	-	5,920
neo-Y-ancY	block1	Tm_014	23,415,924	23,422,199	+	6,276
neo-Y-ancY	block1	Tm_015	24,774,364	24,780,714	+	6,351
neo-Y-ancY	block1	Tm_016	24,848,438	24,854,774	-	6,337
neo-Y-ancY	block1	Tm_017	25,439,702	25,446,038	+	6,337
neo-Y-ancY	block2	Tm_018	30,031,779	30,038,113	+	6,335
neo-Y-ancY	block2	Tm_019	30,616,485	30,622,785	+	6,301
neo-Y-ancY	block2	Tm_020	31,034,442	31,040,793	-	6,352
neo-Y-ancY	block2	Tm_021	31,413,238	31,419,557	+	6,320
neo-Y-ancY	block3	Tm_022	40,473,052	40,479,396	+	6,345
neo-Y-ancY	block3	Tm_023	40,520,103	40,526,439	-	6,337
neo-Y-ancY	block3	Tm_024	40,867,110	40,873,424	+	6,315
neo-Y-ancY	block3	Tm_025	40,914,143	40,920,498	-	6,356
neo-Y-ancY	block3	Tm_026	41,260,993	41,267,291	+	6,299
neo-Y-ancY	block3	Tm_027	41,308,000	41,314,355	-	6,356
neo-Y-ancY	block4	Tm_028	48,736,256	48,742,563	-	6,308
neo-Y-ancY	block4	Tm_029	50,738,674	50,744,997	-	6,324
neo-Y-ancY	block4	Tm_030	51,273,437	51,279,767	-	6,331
neo-Y-ancY	block4	Tm_031	51,641,617	51,647,931	+	6,315
neo-Y-ancY	block4	Tm_032	52,042,675	52,048,984	-	6,310
neo-Y-ancY	block4	Tm_033	52,553,880	52,560,222	+	6,343
neo-Y-ancY	block4	Tm_034	52,628,455	52,634,784	-	6,330
neo-Y-ancY	block5	Tm_035	58,178,691	58,185,022	-	6,332
neo-Y-ancY	block5	Tm_036	58,326,464	58,332,622	+	6,159
neo-Y-ancY	block5	Tm_037	58,344,363	58,350,656	-	6,294
neo-Y-ancY	block5	Tm_038	59,367,068	59,373,352	+	6,285
neo-Y-ancY	block5	Tm_039	59,384,909	59,391,051	-	6,143
neo-Y-ancY	block5	Tm_040	59,709,681	59,715,996	+	6,316
neo-Y-ancY	block5	Tm_041	59,727,788	59,733,947	-	6,160
neo-Y-ancY	block5	Tm_042	60,739,385	60,745,692	+	6,308
neo-Y-ancY	block5	Tm_043	60,757,510	60,763,661	-	6,152
neo-Y-ancY	block5	Tm_044	61,432,105	61,438,253	+	6,149
neo-Y-ancY	block5	Tm_045	62,193,355	62,199,489	-	6,135
neo-Y-ancY	block5	Tm_046	62,882,071	62,888,208	+	6,138

neo-Y-ancY	block5	Tm_047	62,900,037	62,906,351	-	6,315
neo-Y-ancY	block5	Tm_048	64,199,166	64,205,465	+	6,300
neo-Y-ancY	block5	Tm_049	64,217,269	64,223,410	-	6,142
neo-Y-ancY	block5	Tm_050	64,999,334	65,005,468	-	6,135
neo-Y-ancY	block5	Tm_051	65,967,022	65,973,331	+	6,310
neo-Y-ancY	block5	Tm_052	65,985,176	65,991,311	-	6,136
neo-Y-ancY	block5	Tm_053	66,674,203	66,680,354	+	6,152
neo-Y-ancY	block5	Tm_054	66,692,161	66,698,485	-	6,325
neo-Y-ancY	block5	Tm_055	67,683,666	67,689,983	+	6,318
neo-Y-ancY	block5	Tm_056	67,701,749	67,707,886	-	6,138
neo-Y-ancY	block5	Tm_057	68,178,471	68,184,770	+	6,300
neo-Y-ancY	block5	Tm_058	68,196,516	68,202,683	-	6,168
neo-Y-ancY	block5	Tm_059	68,344,425	68,350,755	+	6,331
neo-Y-ancY	block6	Tm_060	73,723,862	73,730,202	+	6,341
neo-Y-ancY	block6	Tm_061	73,798,560	73,804,881	-	6,322
neo-Y-ancY	block6	Tm_062	74,233,616	74,239,947	+	6,332
neo-Y-ancY	block6	Tm_063	74,251,804	74,257,976	-	6,173
neo-Y-ancY	block6	Tm_064	74,760,759	74,766,889	+	6,131
neo-Y-ancY	block6	Tm_065	74,778,784	74,785,097	-	6,314
neo-Y-ancY	block6	Tm_066	75,215,472	75,221,792	+	6,321
neo-Y-ancY	block6	Tm_067	75,290,063	75,296,397	-	6,335
neo-Y-ancY	block7	Tm_068	80,584,335	80,590,665	-	6,331
neo-Y-ancY	block7	Tm_069	80,732,353	80,738,519	+	6,167
neo-Y-ancY	block7	Tm_070	80,750,277	80,756,594	-	6,318
neo-Y-ancY	block7	Tm_071	81,340,669	81,346,977	+	6,309
neo-Y-ancY	block7	Tm_072	81,357,353	81,363,511	-	6,159
neo-Y-ancY	block7	Tm_073	81,651,908	81,658,256	+	6,349
neo-Y-ancY	block7	Tm_074	82,194,149	82,200,471	-	6,323
neo-Y-ancY	block7	Tm_075	82,373,612	82,379,762	+	6,151
neo-Y-ancY	block7	Tm_076	82,391,538	82,397,843	-	6,306
neo-Y-ancY	block7	Tm_077	82,811,960	82,818,293	+	6,334
neo-Y-ancY	block7	Tm_078	83,489,144	83,495,465	+	6,322
neo-Y-ancY	block7	Tm_079	83,507,239	83,513,202	-	5,964
neo-Y-ancY	block7	Tm_080	84,421,292	84,427,583	+	6,292
neo-Y-ancY	block7	Tm_081	84,439,412	84,445,558	-	6,147
neo-Y-ancY	block7	Tm_082	84,621,401	84,627,739	+	6,339
neo-Y-ancY	block7	Tm_083	85,167,691	85,174,034	-	6,344
neo-Y-ancY	block7	Tm_084	85,800,021	85,806,358	+	6,338
neo-Y-ancY	block7	Tm_085	86,944,134	86,950,442	+	6,309
neo-Y-ancY	block7	Tm_086	86,962,239	86,968,374	-	6,136
neo-Y-ancY	block7	Tm_087	87,146,085	87,152,383	+	6,299
neo-Y-ancY	block7	Tm_088	87,692,549	87,698,862	-	6,314
neo-Y-ancY	block7	Tm_089	88,037,478	88,043,797	-	6,320
neo-Y-ancY	block7	Tm_090	88,529,556	88,535,900	-	6,345
neo-Y-ancY	block7	Tm_091	89,145,823	89,152,139	+	6,317
neo-Y-ancY	block7	Tm_092	89,497,069	89,503,376	-	6,308
neo-Y-ancY	block8	Tm_093	122,629,478	122,635,812	+	6,335
neo-Y-ancY	block8	Tm_094	123,118,205	123,124,556	-	6,352
neo-Y-ancY	block8	Tm_095	123,456,782	123,463,123	-	6,342
neo-Y-ancY	block8	Tm_096	124,093,571	124,099,901	+	6,331

neo-Y-ancY	block8	Tm_097	124,250,942	124,257,274	-	6,333
neo-Y-ancY	block9	Tm_098	141,037,741	141,044,061	+	6,321
neo-Y-ancY	block9	Tm_099	141,327,893	141,334,250	-	6,358
neo-Y-ancY	block9	Tm_100	141,968,297	141,974,592	+	6,296
neo-Y-ancY	block9	Tm_101	142,335,226	142,341,588	+	6,363
neo-Y-ancY	block9	Tm_102	142,354,195	142,360,341	-	6,147
neo-Y-ancY	block9	Tm_103	142,495,566	142,501,724	+	6,159
neo-Y-ancY	block9	Tm_104	143,049,510	143,055,813	+	6,304
neo-Y-ancY	block9	Tm_105	143,067,787	143,073,928	-	6,142
neo-Y-ancY	block9	Tm_106	143,258,297	143,264,631	+	6,335
neo-Y-ancY	block9	Tm_107	143,792,304	143,798,617	-	6,314
neo-Y-ancY	block10	Tm_108	170,469,140	170,475,507	-	6,368

Supplementary figures

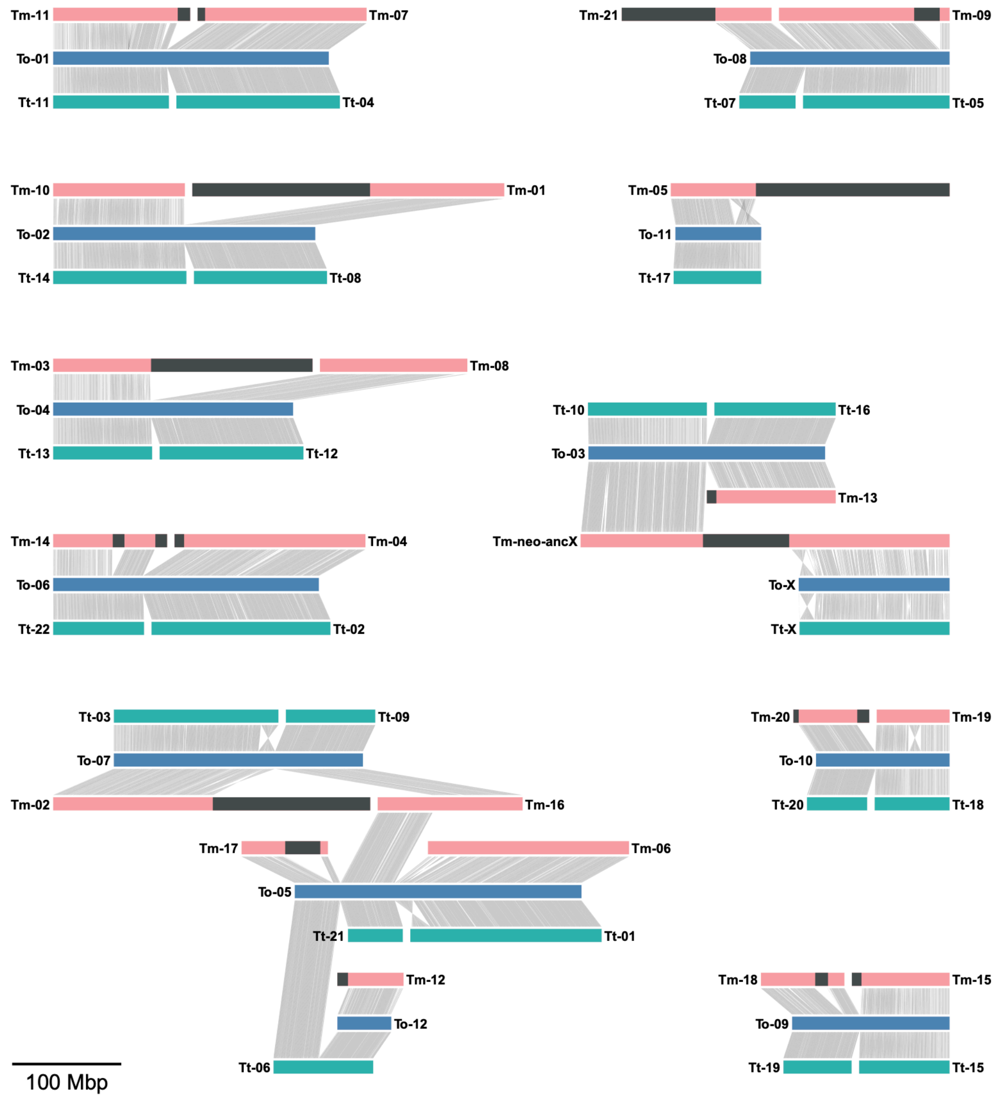

Supplementary Fig. 1. Comparative genomic analysis of the three *Tokudaia* species

Chromosomal-level synteny relationship among the three species. Chromosomes of *T. muenninki* (Tm) is shown in pink, *T. osimensis* (To) in blue, and *T. tokunoshimensis* (Tt) in green, with chromosome numbers indicated. Black-shaded areas represent heterochromatic regions specific to *T. muenninki*. Gray lines between species indicate syntenic relationships. While some inversions were observed, the overall syntenic structure is conserved at the chromosomal level.

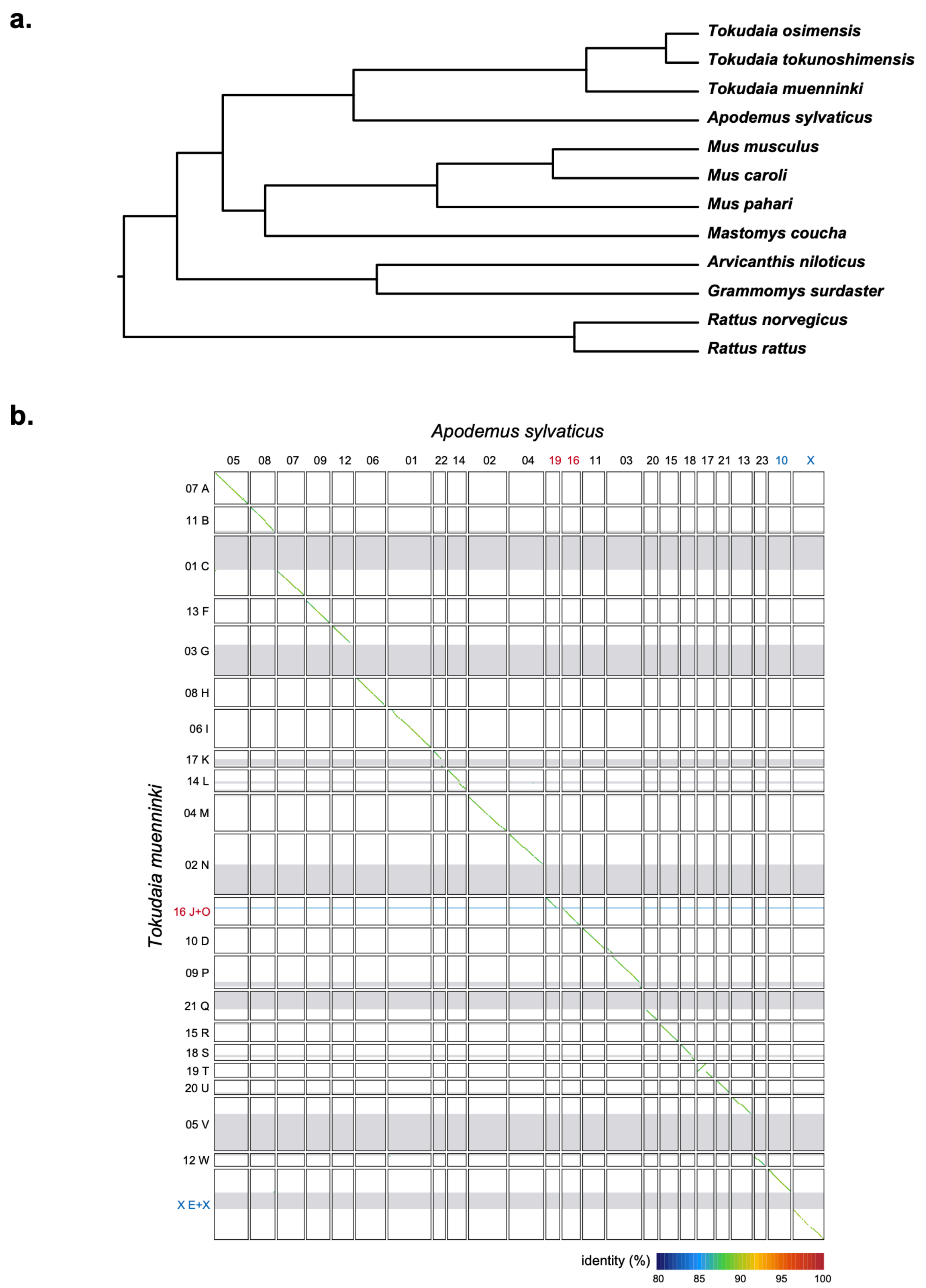

Supplementary Fig. 2. Phylogenetic context and synteny analysis for inferring the ancestral *Tokudaia* karyotype

a Phylogenetic relationship among rodent species with available chromosome-level genome assemblies.

b Synteny dot-plot between *T. muenninki* and *A. sylvaticus*, a closely related outgroup species, illustrating chromosomal fusion events. The results show that fusions of J+O and E+X chromosomes occurred in *T. muenninki*, supporting the inference of the ancestral karyotype of *Tokudaia*.

a.

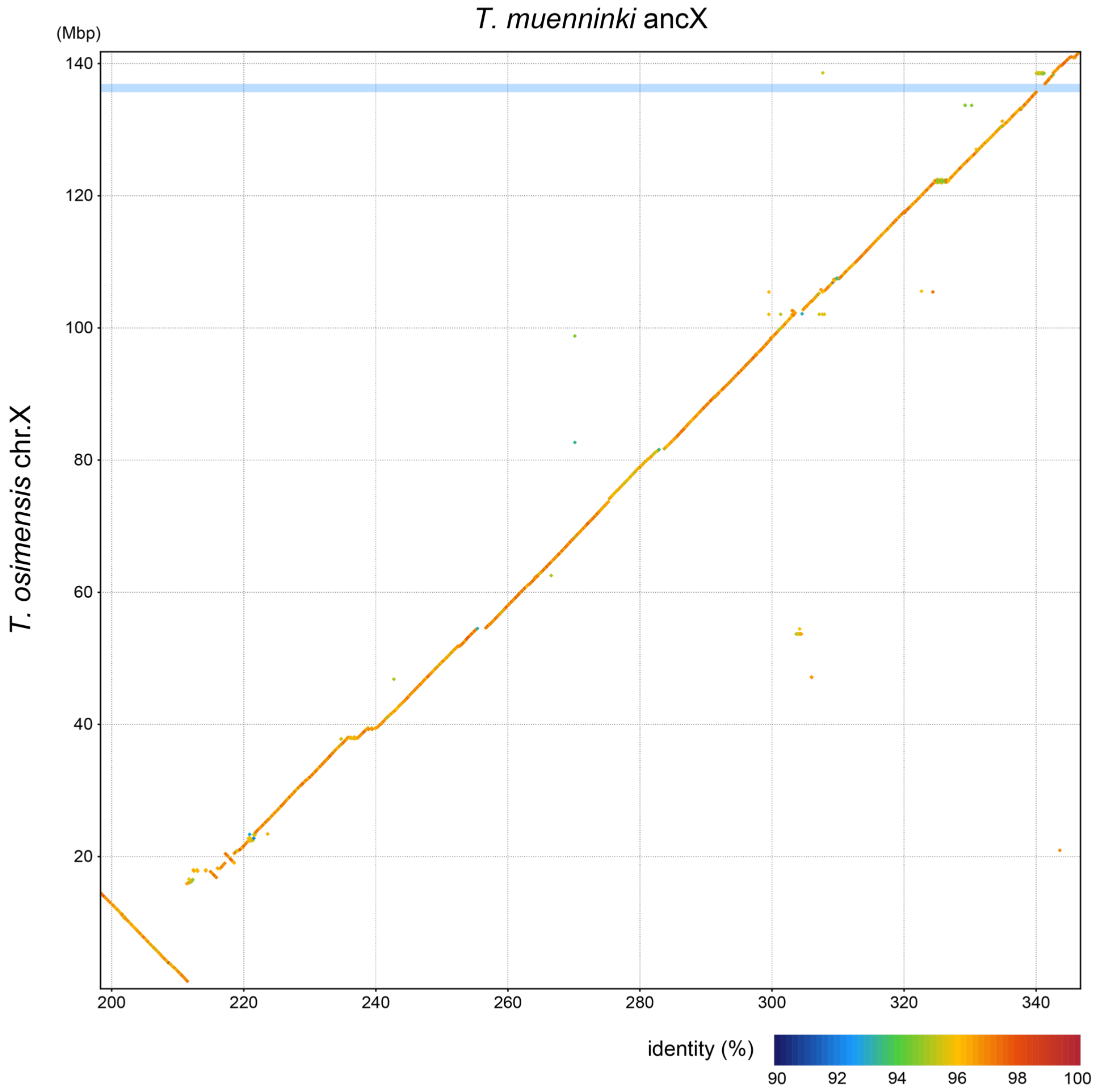

T. muenninki ancX
(Mbp)
140
120
100
80
60
40
20
T. osimensis chr.X
200
220
240
260
280
300
320
340
identity (%)
90
92
94
96
98
100

b.

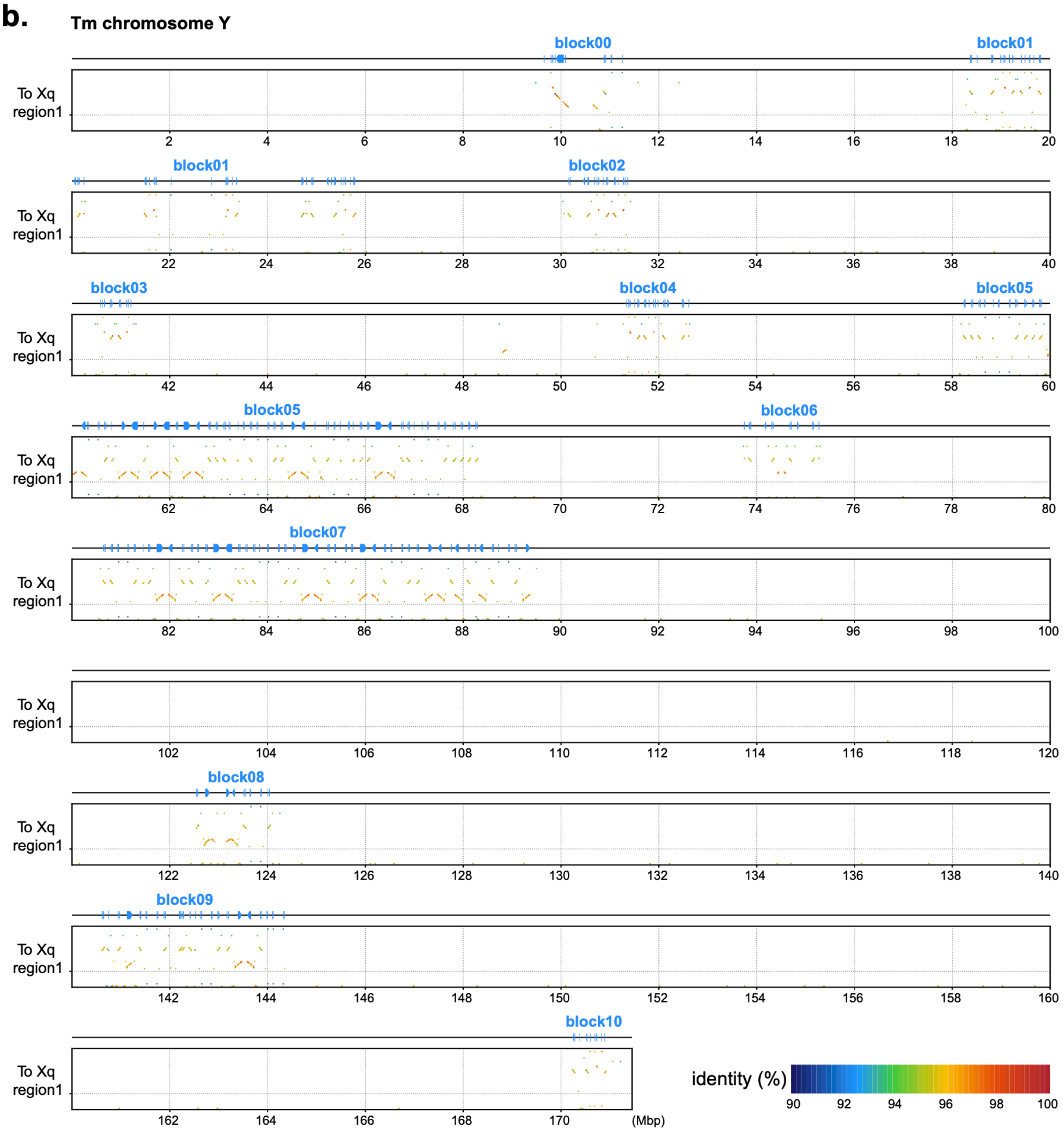

Supplementary Fig. 3. Dot-plot comparison of the sex chromosomes between *T. osimensis* and *T. muenninki*

a Comparison between the full length of the *T. osimensis* X chromosome and the full length of the *T. muenninki* ancX region. The region corresponding to *T. osimensis* Xq-region1 is highlighted in light blue. Extensive synteny is observed across the entire region.

b Comparison between *T. osimensis* Xq-region1 and the full length of the *T. muenninki* Y chromosome.

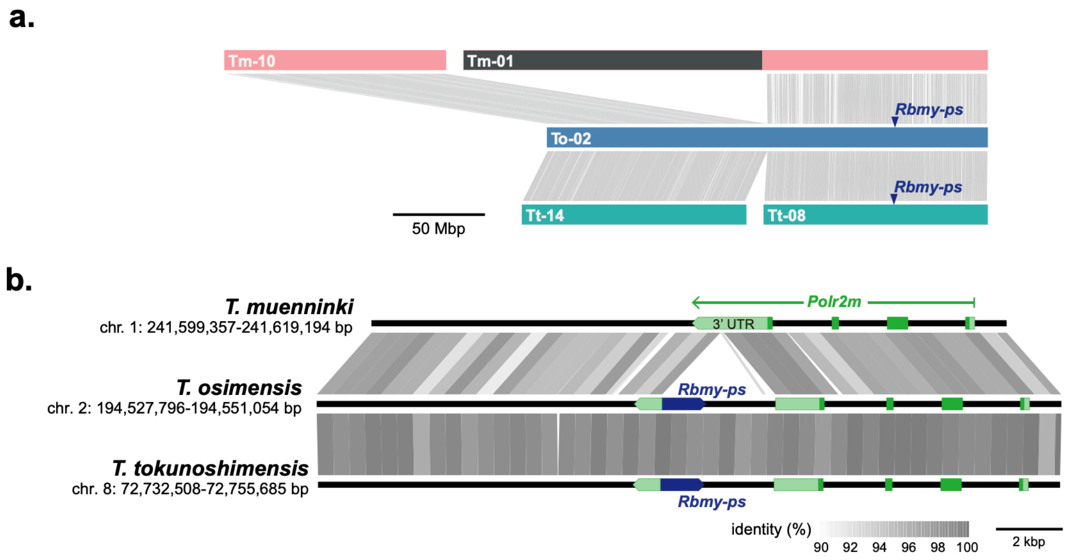

Supplementary Fig. 4 Synteny comparison of the genomic regions surrounding the insertion loci of *Rbmy* processed pseudogenes located on the autosomes in *T. osimensis* and *T. tokunoshimensis*

a A chromosome-scale synteny map showing the location of the *Rbmy* pseudogene insertion. Arrows indicate the insertion points on chromosome 2 in *T. osimensis* and chromosome 8 in *T. tokunoshimensis*. Synteny between the two species suggests that the insertion occurred in a common ancestor.

b A close-up view of the *Rbmy* pseudogene insertion loci. The insertion is absent in *T. muenninki*. It is confirmed that the processed pseudogene is inserted within the 3′-UTR of *Polr2m* in *T. osimensis* and *T. tokunoshimensis*.

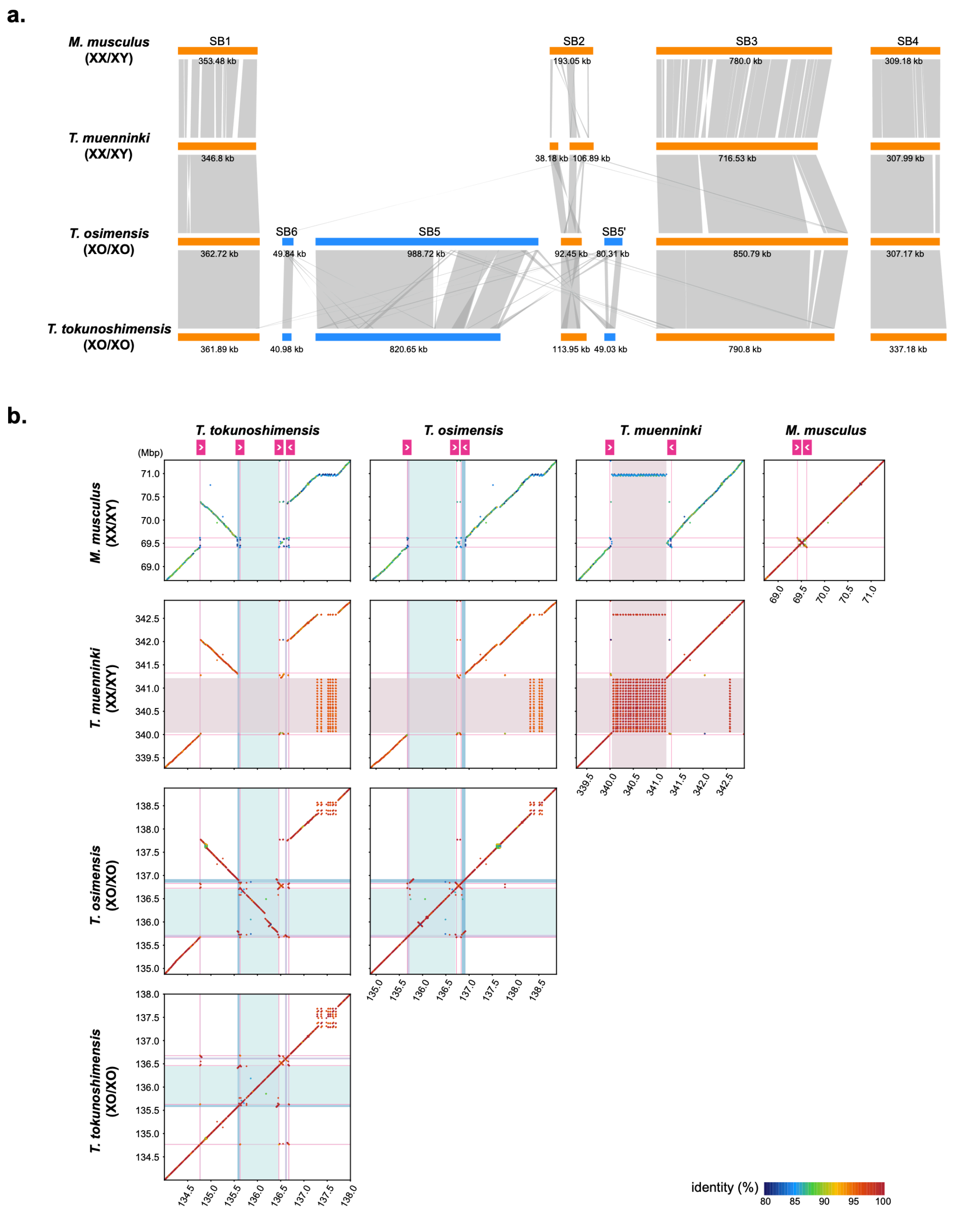

Supplementary Fig. 5. Synteny relationships between mouse and the three *Tokudaia* species around Xq-region1

a Linear alignment diagrams showing synteny blocks (SBs) as defined in *M. musculus*, *T. muenninki*, *T. osimensis*, and *T. tokunoshimensis*. The specific positions of these SBs on each species' X chromosome are detailed in Supplementary Table 1.

b Dot-plot alignments between regions where the *Tokudaia* Y-MRCA region was translocated

to Xq-region1 in *T. osimensis* and *T. tokunoshimensis*. The SB5, SB5′, and SB6 in *T. osimensis* and *T. tokunoshimensis,* along with SB7 in *T. muenninki* are highlighted in light blue, blue, gray, and purple, respectively, and the BASDs are shown in pink.

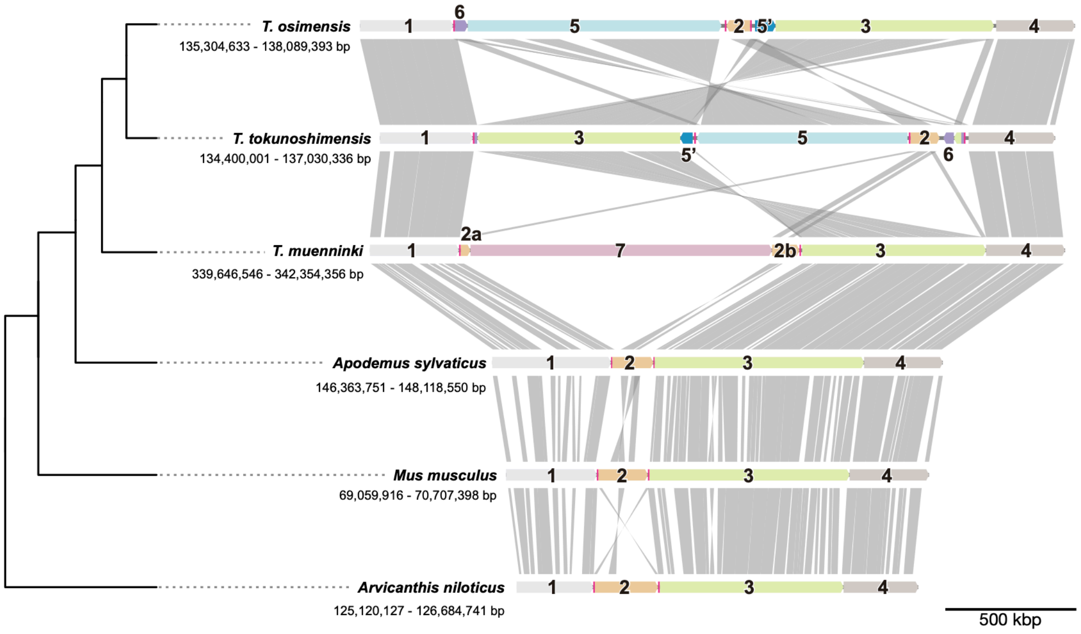

Supplementary Fig. 6. Synteny conservation of SB1-SB4 among *Apodemus sylvaticus*, *Mus musculus*, and *Arvicanthis niloticus*

Alignment of the SB1-SB4 regions among *Apodemus sylvaticus*, *Mus musculus*, and *Arvicanthis niloticus*, showing the conservation of synteny across these rodent species. *A. sylvaticus*, which is phylogenetically closer to *Tokudaia*, exhibits the same arrangement as *M. musculus*, while *A. niloticus*, as an outgroup, also retains this structure. These results support the designation of the SB1-SB4 arrangement of *M. musculus* as ancestral, as it is conserved across multiple rodent lineages.

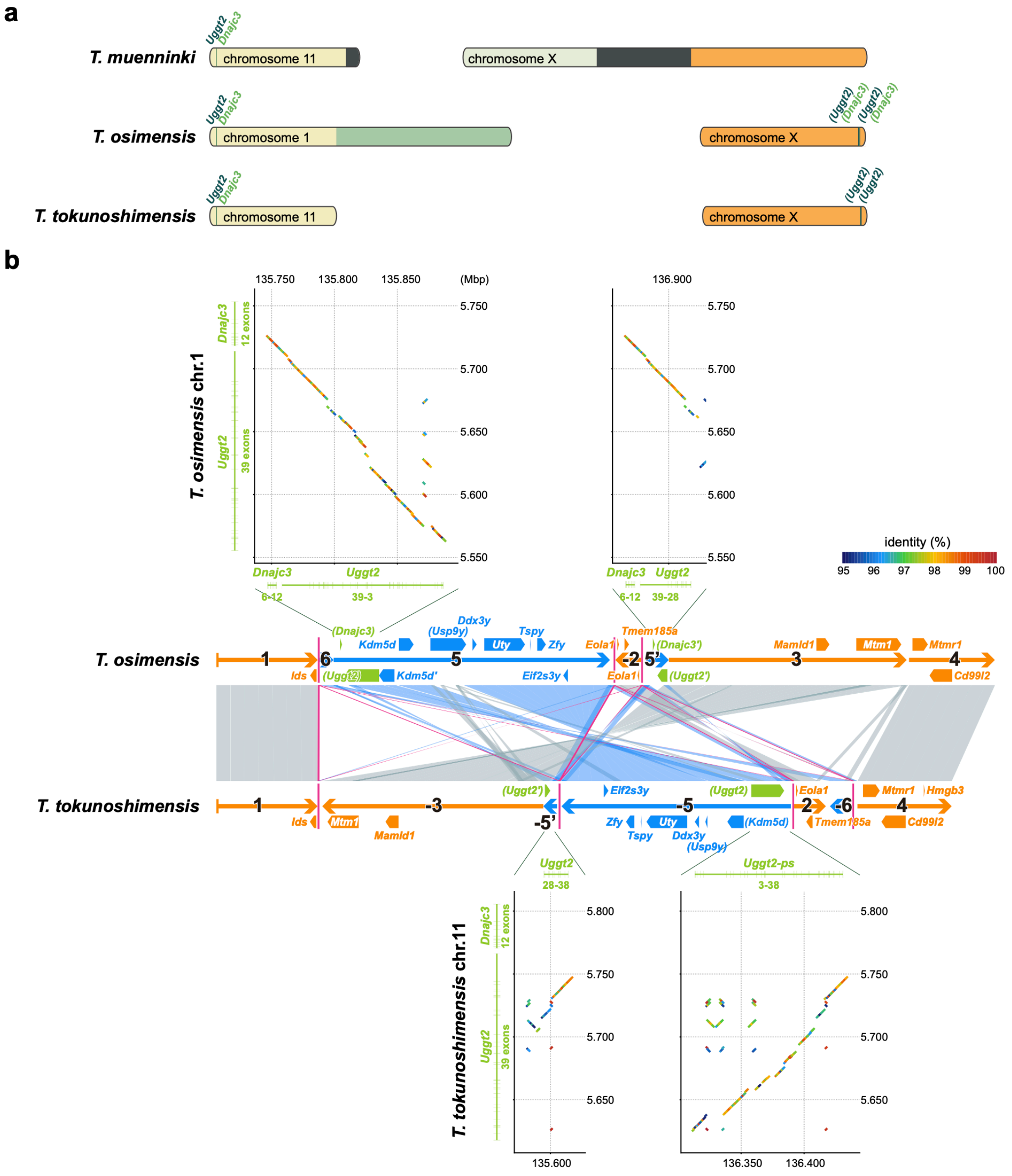

Supplementary Fig. 7. Locations of autosomal-derived genes *Uggt2* and *Dnajc3* found in Xq-region1 across the genome

a Genomic loci of *Uggt2* and *Dnajc3* in the three *Tokudaia* species. In *T. muenninki*, both genes are found only on chromosome 11, whereas in *T. osimensis*, they are located in two positions within Xq-region1, in addition to chromosome 1 (the syntenic region found in *T. muenninki*). In *T. tokunoshimensis*, these genes are found in one location within Xq-region1, as well as on chromosome 11.

b Dot-plot alignment between the surrounding genomic regions of *Uggt2* and *Dnajc3* within Xq-region1 and their corresponding regions on autosomes. The alignment confirms that the autosomal genomic region containing both genes (partially) was translocated into Xq-region1.

a.

the isertion position	ID	the structure of the insertion sequence.						number of inversions		
								T.osi	*T.tok*	total
	12-5060	SB1	SB5	SB6	SB2	SB3	SB4	3	4	7
	12-5061	SB1	SB5	-SB6	SB2	SB3	SB4	3	3	6
between SB1 and SB2	12-5160	SB1	-SB5	SB6	SB2	SB3	SB4	3	4	7
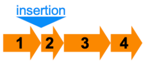	12-5161	SB1	-SB5	-SB6	SB2	SB3	SB4	2	4	6
	12-6050	SB1	SB6	SB5	SB2	SB3	SB4	1	3	4
	12-6051	SB1	SB6	-SB5	SB2	SB3	SB4	2	2	4
	12-6150	SB1	-SB6	SB5	SB2	SB3	SB4	2	4	6
	12-6151	SB1	-SB6	-SB5	SB2	SB3	SB4	3	3	6
	23-5060	SB1	SB2	SB5	SB6	SB3	SB4	3	4	7
	23-5061	SB1	SB2	SB5	-SB6	SB3	SB4	2	4	6
between SB2 and SB3	23-5160	SB1	SB2	-SB5	SB6	SB3	SB4	2	3	5
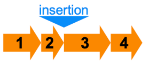	23-5161	SB1	SB2	-SB5	-SB6	SB3	SB4	1	4	5
	23-6050	SB1	SB2	SB6	SB5	SB3	SB4	2	3	5
	23-6051	SB1	SB2	SB6	-SB5	SB3	SB4	3	4	7
	23-6150	SB1	SB2	-SB6	SB5	SB3	SB4	3	2	5
	23-6151	SB1	SB2	-SB6	-SB5	SB3	SB4	3	3	6
	34-5060	SB1	SB2	SB3	SB5	SB6	SB4	3	3	6
	34-5061	SB1	SB2	SB3	SB5	-SB6	SB4	4	2	6
between SB3 and SB4	34-5160	SB1	SB2	SB3	-SB5	SB6	SB4	4	4	8
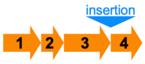	34-5161	SB1	SB2	SB3	-SB5	-SB6	SB4	3	3	6
	34-6050	SB1	SB2	SB3	SB6	SB5	SB4	3	4	7
	34-6051	SB1	SB2	SB3	SB6	-SB5	SB4	4	3	7
	34-6150	SB1	SB2	SB3	-SB6	SB5	SB4	4	4	8
	34-6151	SB1	SB2	SB3	-SB6	-SB5	SB4	3	4	7

b.

12-6050 (*T.osi*: 1, *T. tok*: 3)   12-6051 (*T.osi*: 2, *T. tok*: 2)

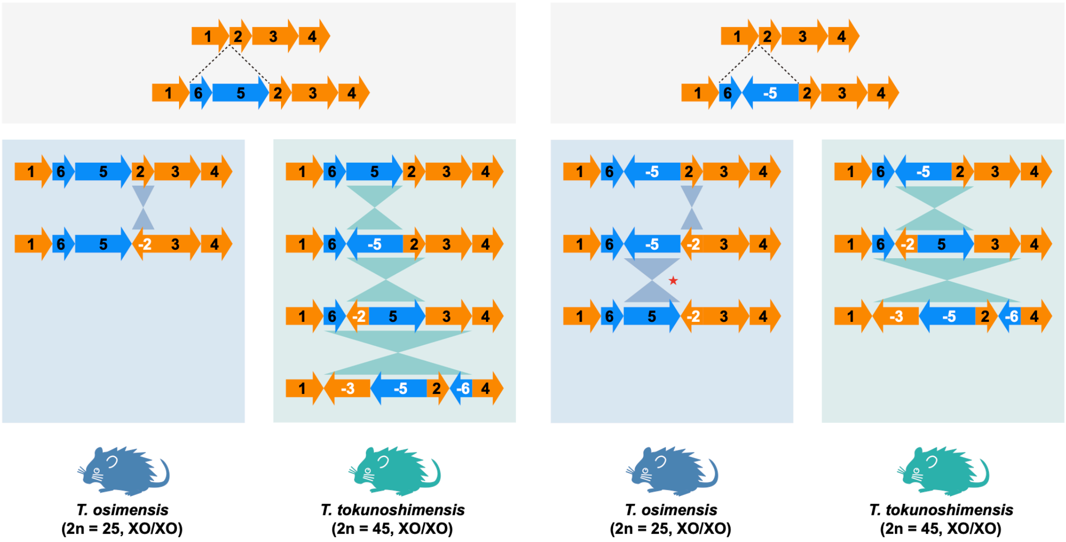

Supplementary Fig. 8. Most parsimonious scenario following the insertion events of SB5 and SB6 into Xq-region1 in *T. osimensis* and *T. tokunoshimensis*

a Estimated insertion positions and directions of SB5 and SB6 with the minimum number of inversions determined by the most parsimonious method. Insertion points considered were between SB1-2, SB2-3, and SB3-4, along with the number of inversions required to achieve the current genomic structure. Two scenarios, highlighted in red, each involve a minimum of four inversions. In both cases, the insertion event likely occurred between SB1 and SB2. For simplicity, SB5 and SB6 are assumed to be inserted as a single continuous block, and SB5′ is omitted from the diagram.

b Two scenarios with the minimum number of inversions [as estimated in **a**.]. In the left scenario, one and three inversions were estimated to have occurred in *T. osimensis* and *T.*

tokunoshimensis, respectively, following the insertion. In the right scenario, two inversions were estimated to have occurred in both *T. osimensis* and *T. tokunoshimensis* following the insertion.

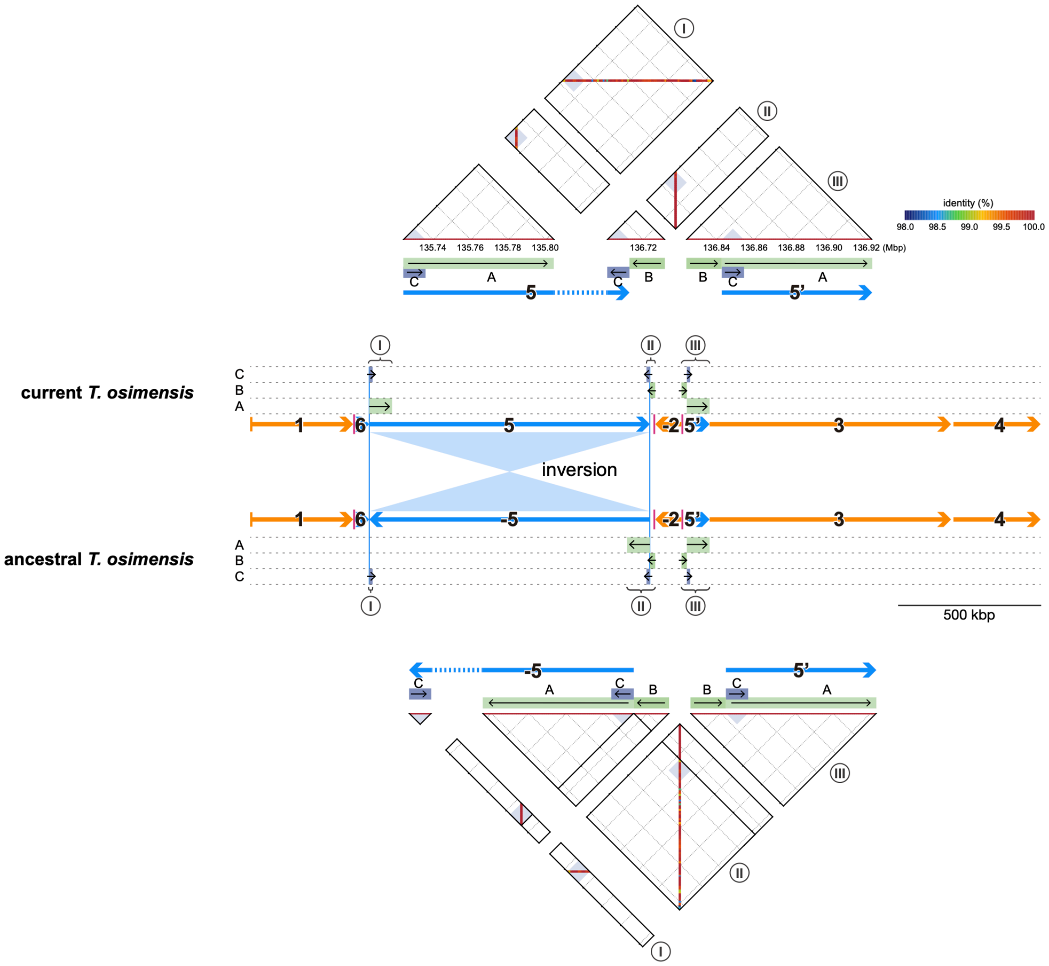

Supplementary Fig. 9. Alignment-based estimation of past inversion events within SB5 in *T. osimensis*

We performed a self-alignment analysis of the SB5-SB2-SB5′ region in *T. osimensis* and identified the following duplicated regions:

- **Region-A (80.3 kb)**: Located in two positions—throughout SB5′ and at the terminal end of SB5 (upper dot-plot, I-III).
- **Region-B (18.9 kb)**: Located in two positions—between SB5 and SB2, and between SB2 and SB5′ (upper dot-plot, II-III) (including BASD).
- **Region-C (12 kb)**: A region largely overlapping the terminal part of Region-A, found in three locations—at the terminal end of SB5′ and at one end of SB5 where Region-A is present, and at the opposite end of SB5 (upper dot-plot, highlighted in purple).

If we assume that SB5 underwent an inversion flanked by Region-C in the past, its original structure before the inversion would have resembled the configuration illustrated in the lower diagram. In this scenario, Region-A and Region-B, currently split and present at opposite ends of SB5 in an inverted orientation, would have been continuous, similar to their

arrangement in SB5′. As shown in the lower dot-plot, Regions II and III form an inverted duplication.

These findings strongly suggest that SB5′ originated from SB5 through an inverted duplication mediated by Region-C, followed by an inversion of SB5 itself. Consequently, the scenario presented in Fig. 3b (Supplementary Fig. 8b, right) is the most plausible.

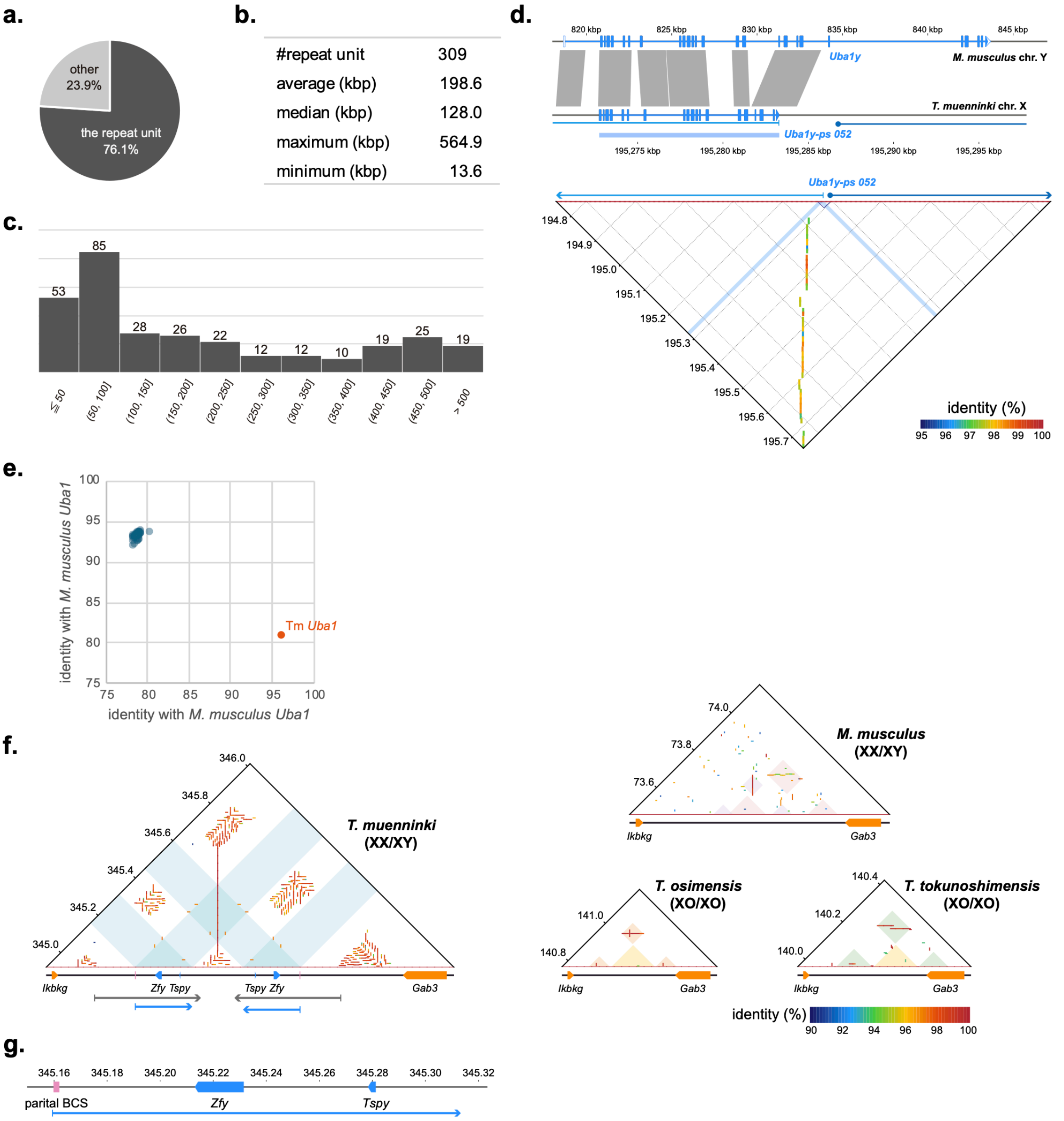

Supplementary Fig. 10. *Tokudaia* Y-MRCA derived region in *T. muenninki* X chromosome

a Breakdown of sequences within the Xhet-region.

b Summary of the repeat sequence unit, the 542 kbp basic unit predominantly making up the Xhet region.

c Length distribution of repeat sequence units, with the 542 kbp as the basic unit.

d Comparison of the *Uba1y* pseudogene (represented by the 52nd pseudogene around 195.3 Mbp) found in the Xhet-region with the intact *Uba1y* gene in *M. musculus*.

The *Uba1y* pseudogenes in the Xhet-region lack the last six exons. The self-dot-plot alignment below confirms the start of the next repeat unit at the position corresponding to these missing exons, ruling out annotation errors.

e Two-dimensional plot depicting the sequence identities of *T. muenninki* genes predicted as *Uba1* and *Uba1y* in comparison to *M. musculus Uba1* and *Uba1y*. *Uba1*, located on the X chromosome, is the gametologous gene of *Uba1y*. Sequence identity was calculated at the nucleotide level using BLASTN. The x-axis represents sequence identity to *M. musculus Uba1*, while the y-axis represents sequence identity to *M. musculus Uba1y*. One gene, highlighted in red, exhibits higher identity to *Uba1* than to *Uba1y*. Genes located in the Xhet-region and the ancY region are shown in blue, all of which exhibit higher identity to *Uba1y* than to *Uba1*, suggesting that they are homologous to *Uba1y*.
f Enlarged view of the self-dot-plot of Xq-region2 (344.9–346.0 Mbp) and corresponding regions in *T. osimensis*, *T. tokunoshimensis*, and *M. musculus*.
For each species, the region between the *Ikbkg* and *Gba3* genes is shown, along with a self-dot-plot alignment diagram. In *T. muenninki*, an inverted duplication of approximately 300 kbp (indicated by the gray arrow) contains the *Tokudaia* Y-MRCA-derived region (indicated by the light blue arrow). In the other three species, no insertion of the Y-chromosome-derived region was observed. However, several tandem and inverted duplications of approximately 100 kbp were detected in this region in each species.
g Enlarged view of the *Tokudaia* Y-MRCA-derived region (one unit of the duplicated region) in *T. muenninki*. This region contains the Y-linked genes (*Zfy* and *Tspy*) and a partial BASD.

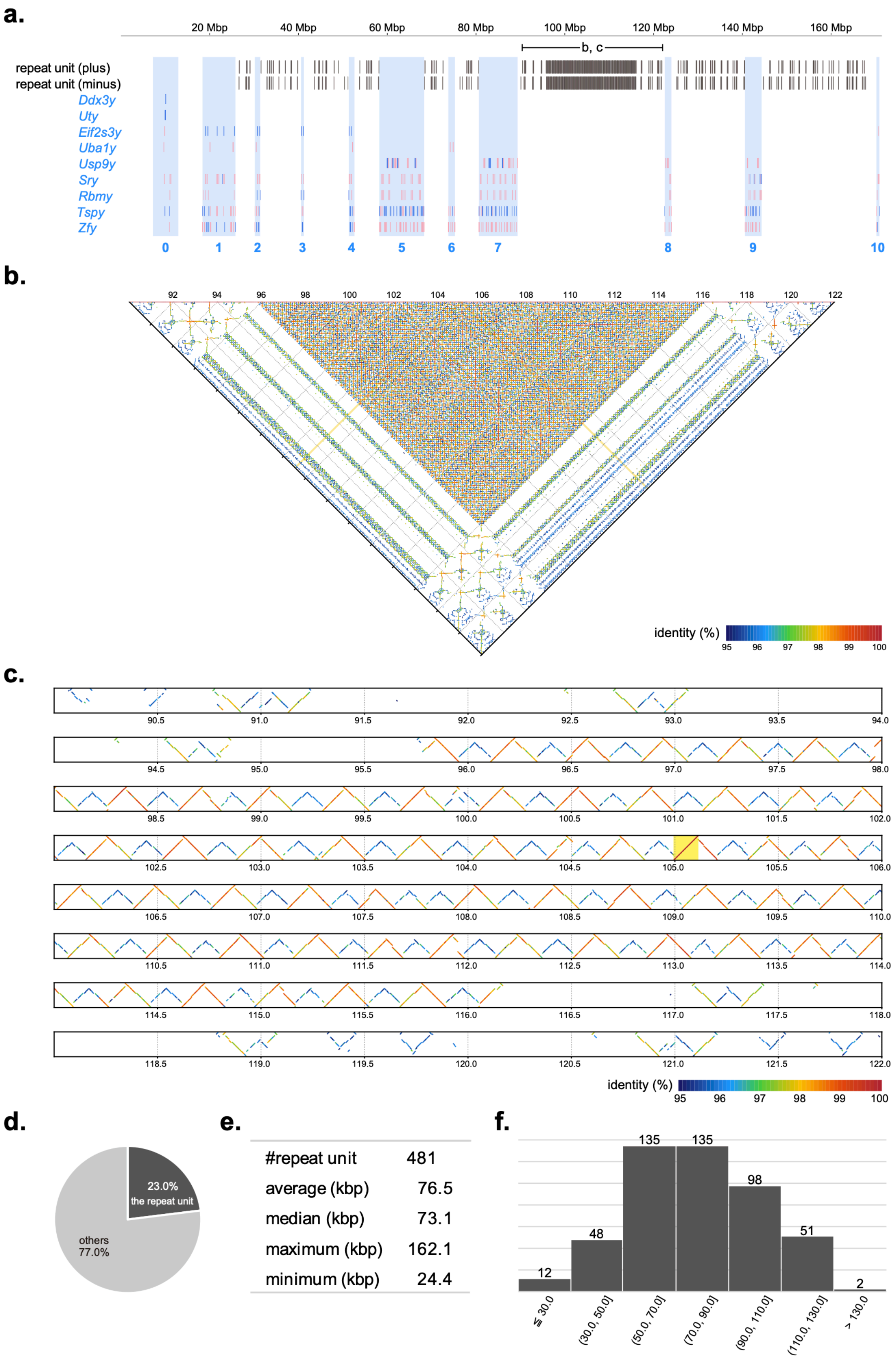

#repeat unit	481
average (kbp)	76.5
median (kbp)	73.1
maximum (kbp)	162.1
minimum (kbp)	24.4

Supplementary Fig. 11. Distribution and characterization of 120 kbp repetitive sequence units in the ancY heterochromatin of *T. muenninki*

a Distribution map of the repetitive sequences with a basic unit of approximately 120 kbp found in the heterochromatin across the entire ancY region. Nine clusters of these sequences were identified.

b Self-dot-plot diagram of the ancY region (90.0–122.0 Mbp), as indicated in a.).

c Dot-plot alignments between the ancY region (x-axis) and the 120 kbp basic unit (y-axis), as indicated in a.). The highlighted yellow square region corresponds to the 120 kbp basic unit.

d Breakdown of the sequences composing the ancY region.

e Summary of the repeat sequence unit, which forms the 120 kbp basic unit primarily in the ancY region.

f Length distribution of the repetitive sequence units (with 120 kbp as the basic unit).

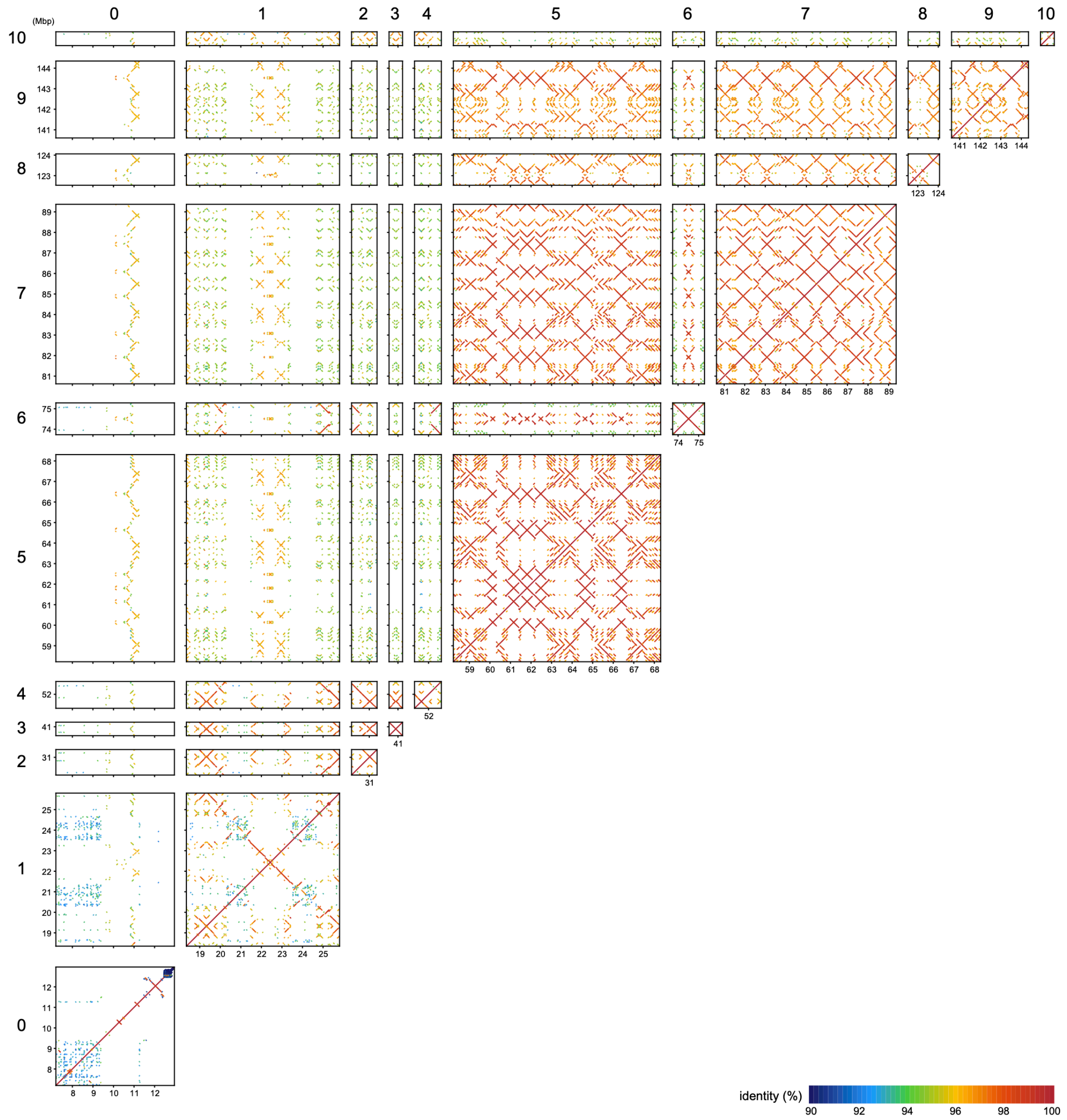

Supplementary Fig. 12. Dot-plot alignment of *Tokudaia* Y-MRCA-derived blocks 0–10 in the ancY region of *T. muenninki*.

High levels of duplication are evident within each block. Blocks 1–4 and Blocks 5–9 show a high degree of homology within each other. In contrast, block 0 exhibits little homology with the other blocks.

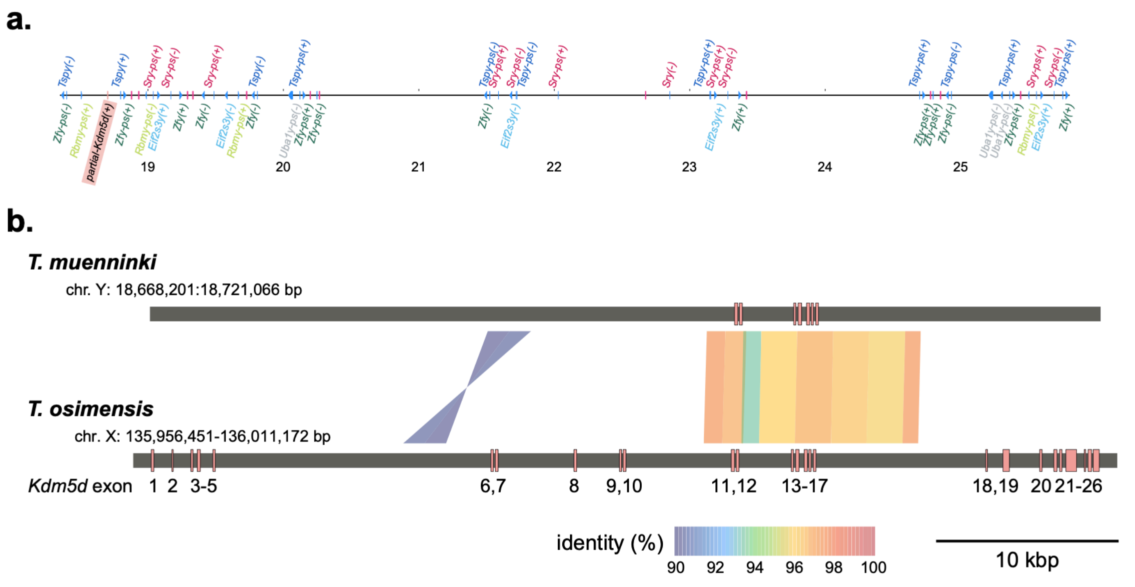

Supplementary Fig. 13. Detailed view of partial *Kdm5d* in the ancY region of *T. muenninki*

a Gene organization of the ancY block 1 region in *T. muenninki*. The partial *Kdm5d* is highlighted in red.

b Enlarged view of the genomic region containing *Kdm5d*, with a comparative alignment between *T. muenninki* and *T. osimensis*. The pink squares represent the exons of *Kdm5d*, and the numbers below each exon indicate the exon numbers. While *T. osimensis* retains all 26 exons of *Kdm5d*, *T. muenninki* possesses only exons 11–17, and alignment is observed only in the surrounding regions.

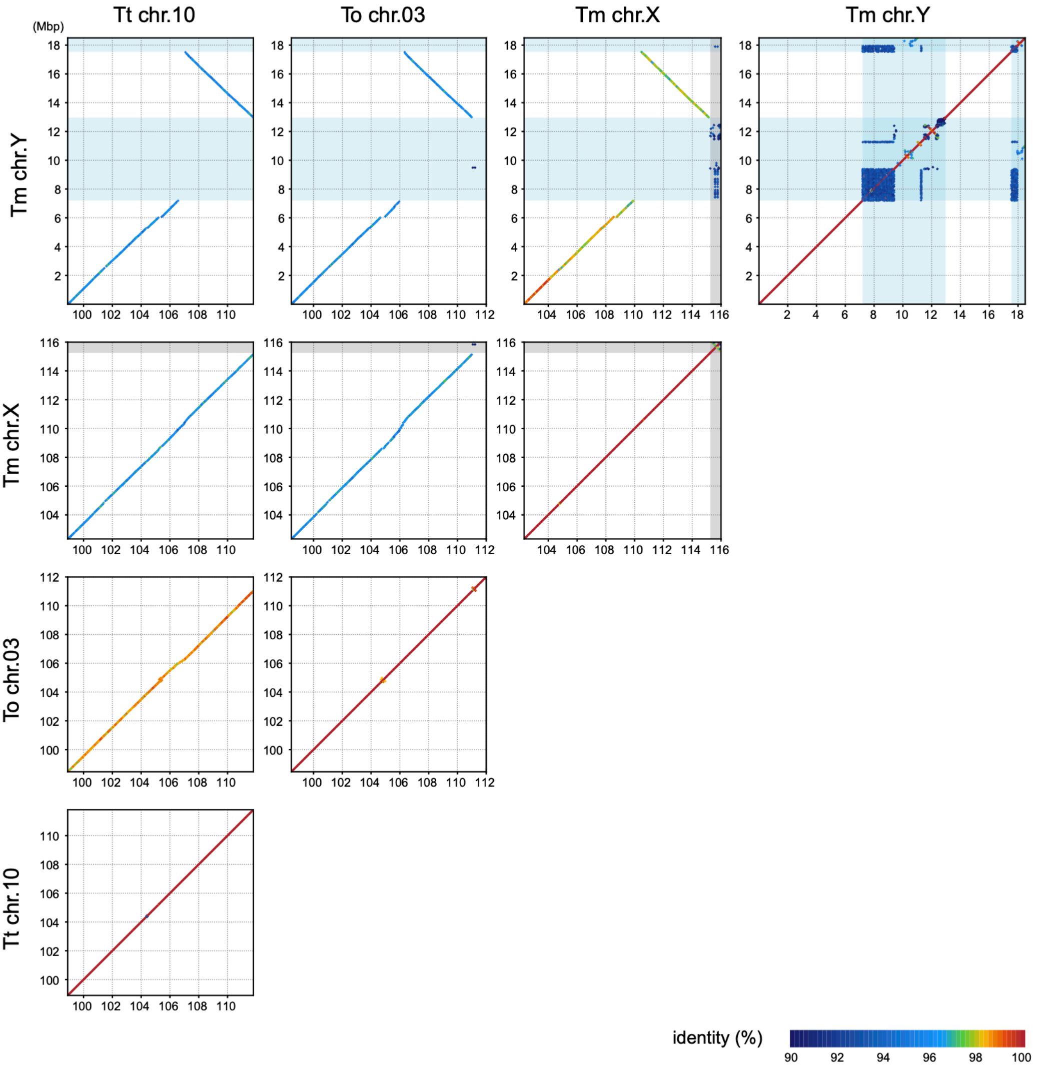

Supplementary Fig. 14. Dot-plot alignment between Strata0 – Strata1a – Strata1b regions in *T. muenninki* (neo regions of both X and Y chromosomes) and corresponding autosomal regions in *T. osimensis* and *T. tokunoshimensis*

The regions highlighted in light blue represent *Tokudaia* Y-MRCA-derived regions: blocks 0 and 1. The alignment shows that the structure on the X chromosome side of *T. muenninki* is also present in *T. osimensis* and *T. tokunoshimensis*. This suggests that an inversion occurred on the neo-Y side in *T. muenninki*.

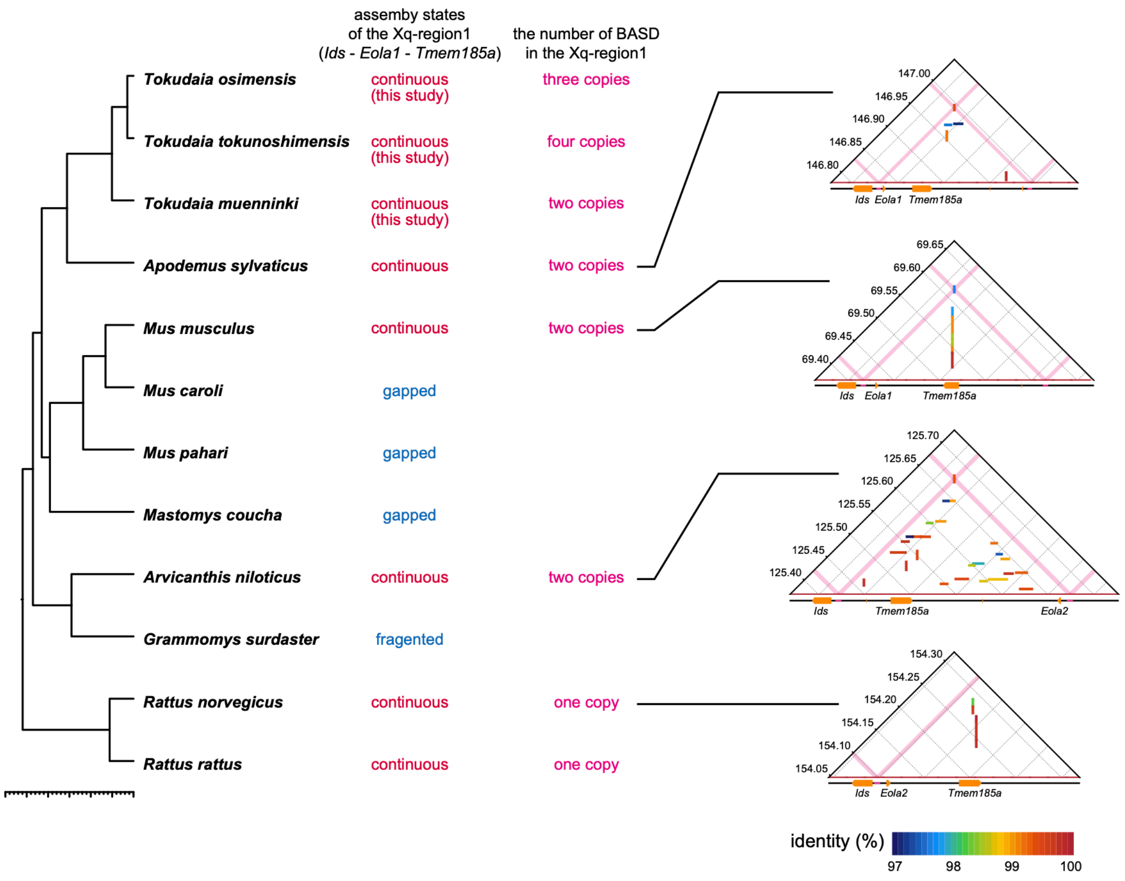

Supplementary Fig. 15. Results of the search for sequences homologous to boundary-associated segmental duplication (BASD) using genomes of nine rodent species, in addition to the three *Tokudaia* species

This figure shows the presence of genomic regions homologous to Xq-region1 and the number of copies of sequences homologous to BASD identified, alongside the phylogenetic tree. Except for the three *Tokudaia* species, only one or two copies of BASD homologous sequences were identified in regions homologous to Xq-region1. Self-dot-plot diagrams of the regions where the sequences were found are also shown, with pink areas indicating the presence of BASD.

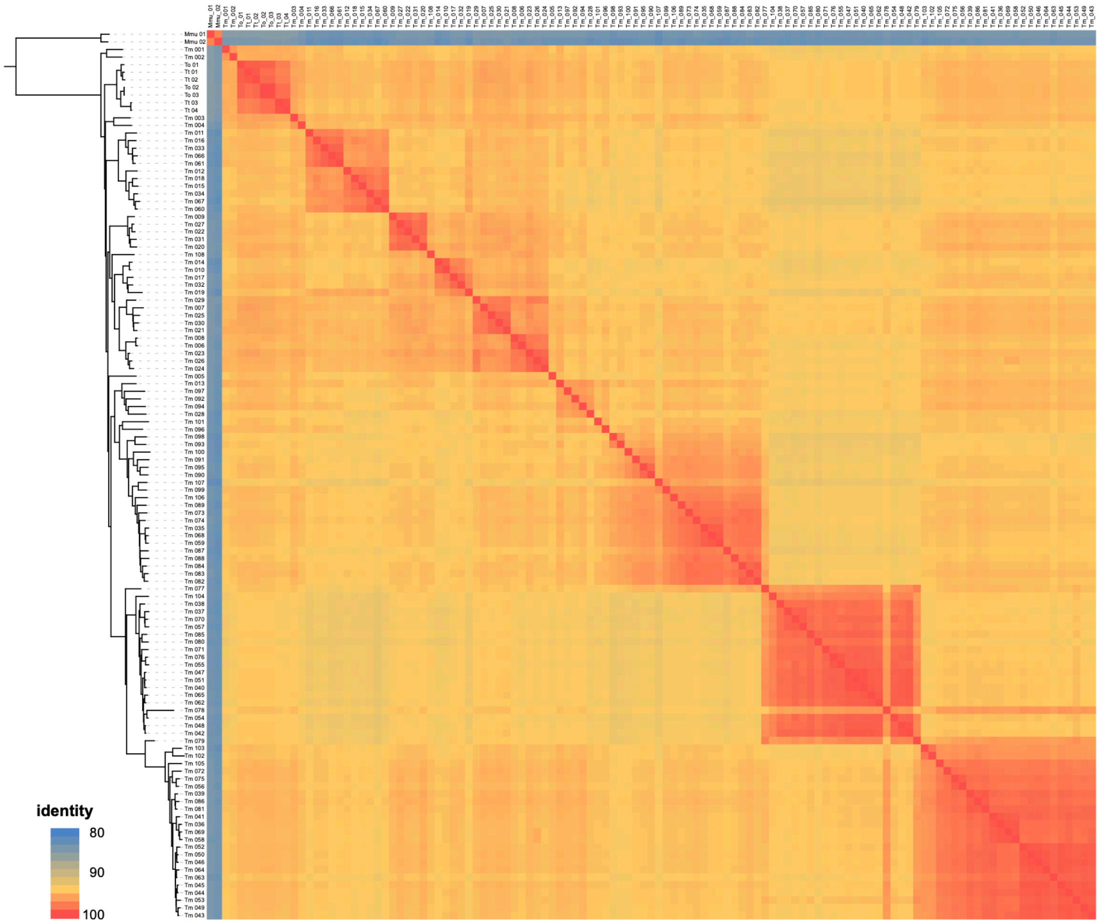

Supplementary Fig. 16. Heatmap of sequence identity among all BASD copies identified in this study

This figure shows pairwise sequence identity between all Boundary-Associated Segmental Duplication (BASD) copies detected in the *Tokudaia* and mouse genomes.

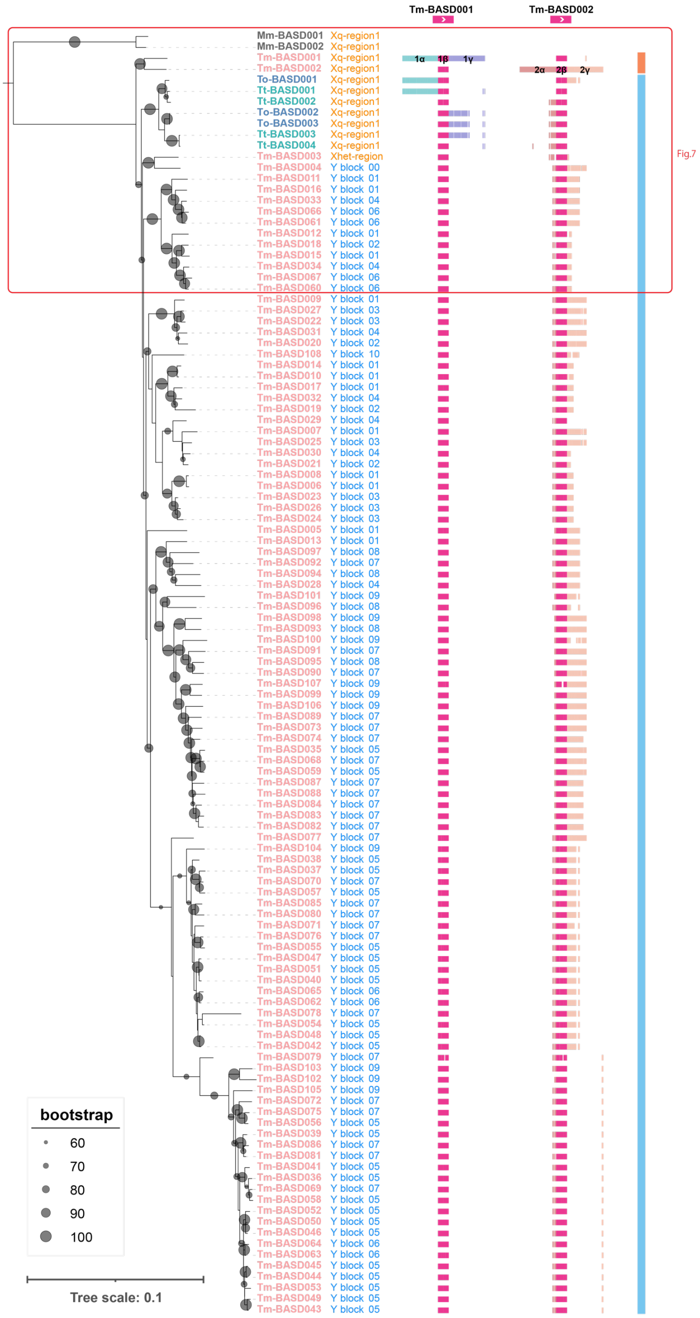

Supplementary Fig. 17. Full tree of the boundary-associated segmental duplications (BASDs), in which the collapsed clade in Fig. 7a is expanded.

Phylogenetic tree illustrates the relationships among all BASDs found in the genomes of the three *Tokudaia* species and the mouse. The tiles on the right represent regions of sequence similarity identified in other BASDs, using Tm-BASD001 and Tm-BASD002 along with their 20 kbp upstream and downstream sequences as references. The red boxes indicate the clades that are expanded in Fig. 7a.

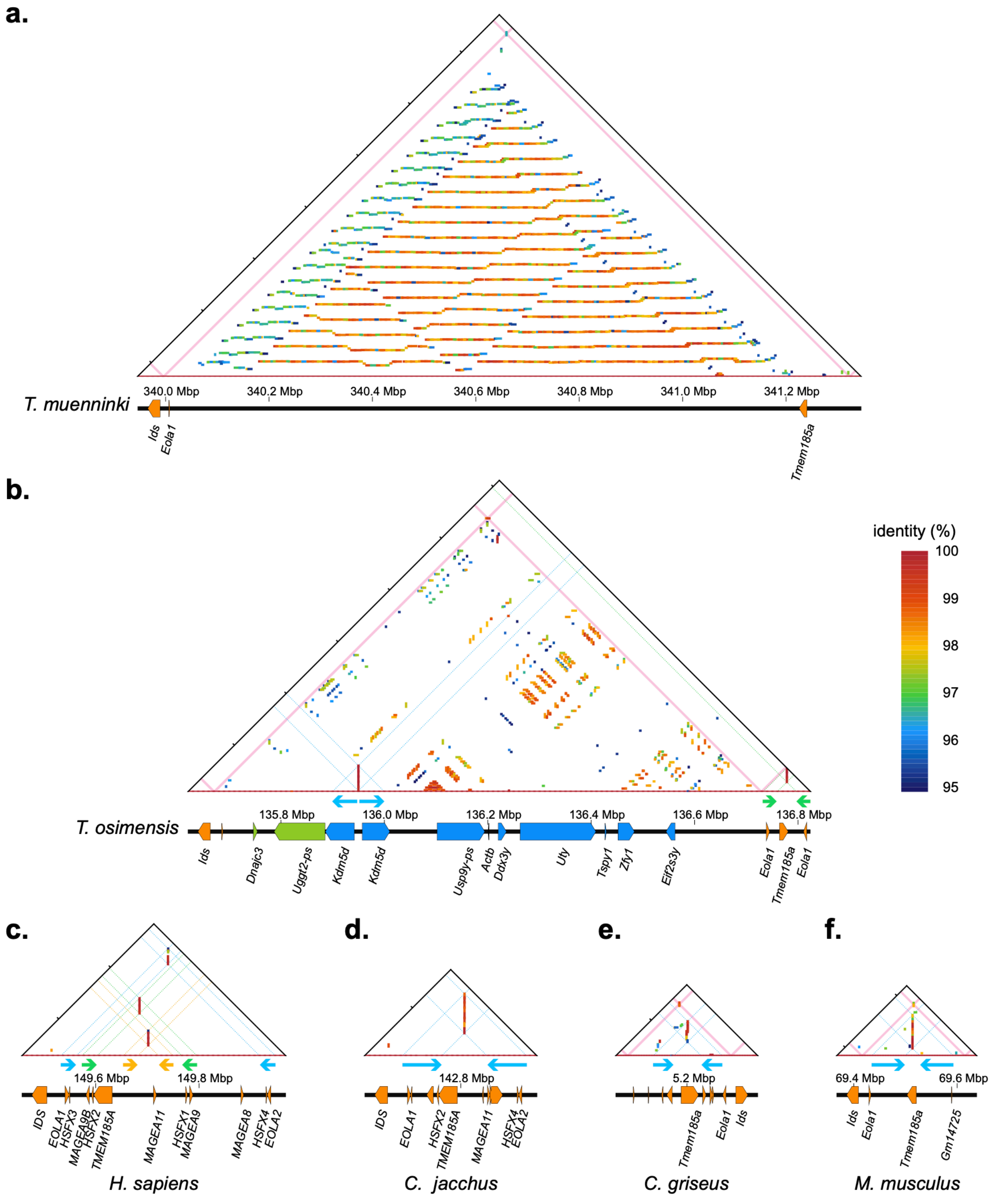

Supplementary Fig. 18. Self-dot-plot alignment of the Xq-region1 corresponding regions — *Ids* – *Eola* region — across various species

Self-dot-plot alignment results of the Xq-region1 in multiple species. Pink-shaded areas indicate BASD, while arrows denote segmental duplications. The colored arrows below the dot-plot indicate the positions and lengths of palindrome structures identified in this locus for each species. **a** *T. muenninki* **b** *T. osimensis* **c** *H. sapiens* **d** *Callithrix jacchus* **e** *Cricetulus griseus* **f** *M. musculus*

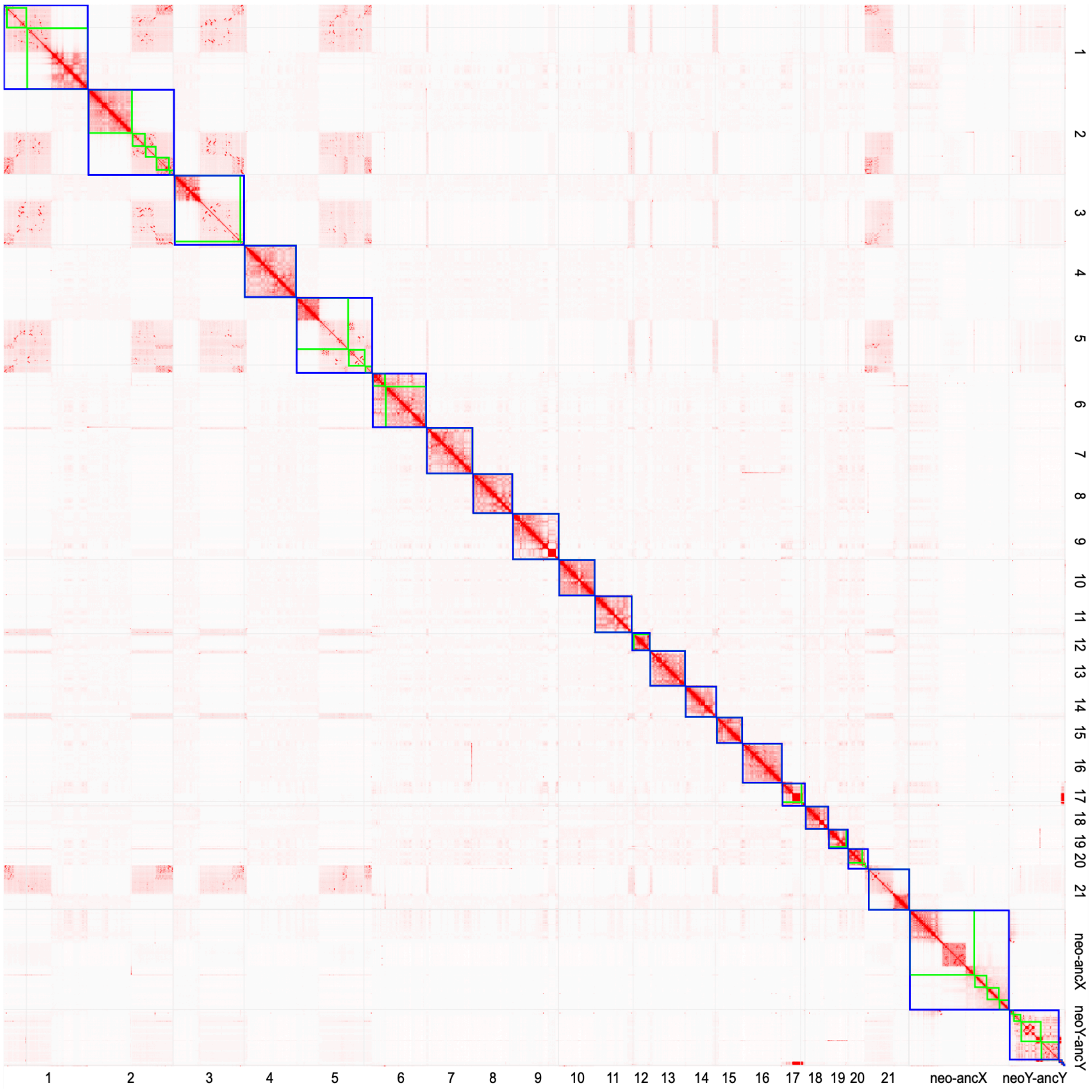

Supplementary Fig. 19. Hi-C contact map of *T. muenninki*

The blue squares represent chromosomes.

Supplementary references

Altschul SF, Gish W, Miller W, Myers EW, Lipman DJ. 1990. Basic local alignment search tool. *J Mol Biol* 215:403–410.

Capella-Gutiérrez S, Silla-Martínez JM, Gabaldón T. 2009. trimAl: a tool for automated alignment trimming in large-scale phylogenetic analyses. *Bioinformatics* 25:1972–1973.

Cosentino S, Iwasaki W. 2019. SonicParanoid: fast, accurate and easy orthology inference. *Bioinformatics* 35:149–151.

Katoh K, Standley DM. 2013. MAFFT Multiple Sequence Alignment Software Version 7: Improvements in Performance and Usability. *Mol Biol Evol* 30:772–780.

Li H. 2018. Minimap2: pairwise alignment for nucleotide sequences. *Bioinformatics* 34:3094–3100.

Nguyen L-T, Schmidt HA, von Haeseler A, Minh BQ. 2015. IQ-TREE: A Fast and Effective Stochastic Algorithm for Estimating Maximum-Likelihood Phylogenies. *Mol Biol Evol* 32:268–274.

Shen W, Le S, Li Y, Hu F. 2016. SeqKit: a cross-platform and ultrafast toolkit for FASTA/Q file manipulation. *PLoS One* 11:e0163962.
